# Rebalancing TGF‐β/Smad7 signaling via Compound kushen injection in hepatic stellate cells protects against liver fibrosis and hepatocarcinogenesis

**DOI:** 10.1002/ctm2.410

**Published:** 2021-07-04

**Authors:** Yang Yang, Mayu Sun, Weida Li, Chaobao Liu, Zheshun Jiang, Pengfei Gu, Jingquan Li, Wei Wang, Rongli You, Qian Ba, Xiaoguang Li, Hui Wang

**Affiliations:** ^1^ State Key Laboratory of Oncogenes and Related Genes, Center for Single‐Cell Omics, School of Public Health Shanghai Jiao Tong University School of Medicine Shanghai China; ^2^ CAS Key Laboratory of Nutrition, Metabolism and Food Safety Shanghai Institute of Nutrition and Health, University of Chinese Academy of Sciences, Chinese Academy of Sciences Shanghai China; ^3^ Beijing Zhendong Pharmaceutical Research Institute Co., Ltd. Beijing China

**Keywords:** Compound kushen injection, hepatocellular carcinoma, liver fibrosis, TGF‐β/Smad signaling, traditional chinese medicine

## Abstract

**Background:**

Liver fibrosis and fibrosis‐related hepatocarcinogenesis are a rising cause for morbidity and death worldwide. Although transforming growth factor‐β (TGF‐β) is a critical mediator of chronic liver fibrosis, targeting TGF‐β isoforms and receptors lead to unacceptable side effect. This study was designed to explore the antifibrotic effect of Compound kushen injection (CKI), an approved traditional Chinese medicine formula, via a therapeutic strategy of rebalancing TGF‐β/Smad7 signaling.

**Methods:**

A meta‐analysis was performed to evaluate CKI intervention on viral hepatitis‐induced fibrosis or cirrhosis in clinical randomized controlled trials (RCTs). Mice were given carbon tetrachloride (CCl_4_) injection or methionine‐choline deficient (MCD) diet to induce liver fibrosis, followed by CKI treatment. We examined the expression of TGF‐β/Smad signaling and typical fibrosis‐related genes in hepatic stellate cells (HSCs) and fibrotic liver tissues by qRT‐PCR, Western blotting, RNA‐seq, immunofluorescence, and immunohistochemistry.

**Results:**

Based on meta‐analysis results, CKI improved the liver function and relieved liver fibrosis among patients. In our preclinical studies by using two mouse models, CKI treatment demonstrated promising antifibrotic effects and postponed hepatocarcinogenesis with improved liver function and histopathologic features. Mechanistically, we found that CKI inhibited HSCs activation by stabilizing the interaction of Smad7/TGF‐βR1 to rebalance Smad2/Smad3 signaling, and subsequently decreased the extracellular matrix formation. Importantly, Smad7 depletion abolished the antifibrotic effect of CKI in vivo and in vitro. Moreover, matrine, oxymatrine, sophocarpine, and oxysophocarpine were identified as material basis responsible for the antifibrosis effect of CKI.

**Conclusions:**

Our results unveil the approach of CKI in rebalancing TGF‐β/Smad7 signaling in HSCs to protect against hepatic fibrosis and hepatocarcinogenesis in both preclinical and clinical studies. Our study suggests that CKI can be a candidate for treatment of hepatic fibrosis and related oncogenesis.

## Graphical abstract

1. CKI ‐suppresses liver fibrosis and hepatocarcinogenesis in both preclinical and clinical studies.

2. CKI inhibits HSCs activation by stabilizing the interaction of Smad7/TGFβR1 to rebalance Smad2/Smad3 signaling, acting as an alternative approach to target TGF‐β signaling.

3. High expression of Smad7 and low expression of TGFβR1 in HCC tumors and surrounding normal liver tissues can be tumor suppressive.

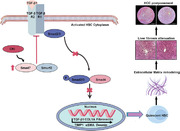

**Abbreviations**
ALT: alanine transaminase
AST: aspartate transaminase
CCl_4_: carbon tetrachloride
CKI: Compound kushen injection
COL1A: collagen type I
COL3: procollagen type III
COL4: collagen type IV
GSEA: gene set enrichment analysis
H&E: hematoxylin and eosin
HA: hyaluronic acid
HCC: hepatocellular carcinoma
HPLC: high‐performance liquid chromatography
HSCs: hepatic stellate cells
IL‐6: interleukin‐6
LN: laminin
MCD: methionine‐choline deficient
MDA: malondialdehyde
NASH: nonalcoholic steatohepatitis
NMPA: National Medical Products Administration
PCNA: proliferating cell nuclear antigen
RCTs: randomized controlled trials
SOD: superoxide dismutase
T‐BIL: total bilirubin
TCM: traditional Chinese medicine
TGF‐β: transforming growth factor‐β
TGFβR1: TGF‐β receptor type 1
TGFβR2: TGF‐β receptor type 2
TIMP1: tissue inhibitor of metalloproteinase 1
TNFα: tumor necrosis factor α
αSMA: α‐smooth muscle actin

## 1 INTRODUCTION

Liver fibrosis, which can progress to cirrhosis and liver cancer, is a primary cause of global morbidity and mortality related to liver diseases.^1^ It is the common pathologic outcome of extended inflammation and damage in liver. There are multiple etiologies related to progressive liver fibrosis and oncogenesis, including chronic viral infection, nonalcoholic fatty liver disease, nonalcoholic steatohepatitis (NASH), alcoholism, autoimmune hepatitis, and biliary diseases.^2^ Liver fibrosis is forcefully linked with hepatocellular carcinoma (HCC), where the incidence of HCC caused by liver cirrhosis reaches up to 90%,^3^ which made liver cirrhosis recognized as the primary risk factor for HCC.^4^ Currently, the solely available treatment for liver fibrosis is elimination of chronic stimulus and/or liver transplantation.^1^ Due to lack of effective therapeutics and the long period of progression from liver fibrosis to HCC,^5^ it is necessary to develop new antifibrotic therapy to attenuate liver fibrosis and reduce the risk of progression toward HCC.

Hepatic stellate cells (HSCs) are a key effector for progressive liver fibrosis, functioning to store retinyl esters in lipid droplets at quiescent status.^6^, ^7^ However, quiescent HSCs are activated and trans‐differentiated into myofibroblasts in the fibrotic liver, which are recognized as the primary origin of extracellular matrix molecules including collagens, α‐smooth muscle actin (αSMA), fibronectin, and tissue inhibitor of metalloproteinase 1 (TIMP1).^8^, ^9^ Transforming growth factor‐β (TGF‐β) plays a vital role in promoting HSCs activation and myofibroblast trans‐differentiation to promote fibrosis progression.^10^ Therefore, restricting HSCs activation and inhibiting TGF‐β signaling can be a promising goal of developing a new antifibrotic therapy. However, in preclinical and clinical studies, direct targeting of TGF‐β axis by neutralizing TGF‐β isoforms or inhibiting TGF‐β receptors leads to unacceptable adverse effects probably due to the diversity of the potential biological context‐dependent roles of TGF‐β.^11^, ^12^ Therefore, greater understanding of how TGF‐β plays a role in liver fibrosis, and developing alternative strategies to interfere with TGF‐β at other levels is needed.

Herbal formulations have been investigated in the treatment of liver fibrosis.^2^ Compound kushen injection (CKI), a typical traditional Chinese medicine (TCM), has been approved to clinically treat cancer‐induced pain in China for over 20 years by Chinese National Medical Products Administration (NMPA).^13^ CKI is extracted from the roots of Kushen and Baituling with several identified bioactive alkaloids, including oxymatrine, matrine, oxysophocarpine, and sophocarpine.^14^ Preclinical studies indicated that CKI exhibited a broad‐spectrum antineoplastic function against gastric cancer, colon cancer, breast cancer, lung carcinoma, and acute myeloid leukemia.^15^, ^16^, ^17^, ^18^, ^19^, ^20^ Our previous research also revealed that CKI could remodel tumor microenvironment to produce a therapeutic effect against HCC.^21^ In recent years, several clinical randomized controlled trials (RCTs) in China showed that CKI could improve symptoms caused by chronic hepatitis B and C, liver fibrosis, and cirrhosis with minimal observed side effects. However, the efficacy and underlying mechanism of CKI on hepatic fibrosis and related oncogenesis remain to be elucidated.

Here, we proposed to study the efficacy of CKI on liver fibrosis and its underlying mechanism. With clinical meta‐analysis and two preclinical animal models, we showed that CKI attenuated chronic liver fibrosis and reduced HCC formation. Importantly, we demonstrated that CKI selectively re‐established the balance between profibrotic Smad2/3 activation and antifibrotic Smad7 action in hepatic stellate cells, acting as an alternative approach to target TGF‐β signaling.

## 2 METHODS

### 2.1 Cell lines and reagents

Human hepatic stellate cell line LX‐2 cells were purchased from Merck Millipore (Darmstadt, Germany). Human hepatic cell line LO2 cells were purchased from American Type Culture Collection (ATCC, USA). The cell lines were routinely tested using mycoplasma contamination kit (R&D) and cultured in DMEM (high glucose) or RPMI 1640 medium complemented with 10% FBS after thawing and cultured at 37°C in a humidified atmosphere containing 5% CO_2_.

All chemicals were of analytical grade. CKI was provided by Shanxi Zhendong Pharmaceutical Co. Ltd (Changzhi, China). The high‐performance liquid chromatography (HPLC) fingerprint of CKI and list of four identified main bioactive alkaloids content in CKI are indicated in Figure S1 and Table S1. Carbon tetrachloride (CCl_4_) and olive oil were purchased from Sinopharm Chemical Reagent Co. Ltd (Shanghai, China). Methionine‐choline deficient (MCD) diet was obtained from Research Diets, Inc. (NJ, USA).

### 2.2 Animal models

C57BL/6 mice (male, 4–8 weeks, Certificate number: SCXK [Shanghai] 2017‐0005) were obtained from Shanghai Slac Laboratory Animal Co. (Shanghai, China) and fed in a pathogen‐free vivarium under standard conditions. Animal protocols were executed in accordance with the SIBS Guide for Care and Use of Laboratory Animals and approved by the Animal Care and Use Committee of Institute for Nutritional and Health, SIBS, CAS (Certificate number: SIBS‐2019‐WH‐1).

To establish CCl_4_‐induced chronic liver fibrosis models, C57BL/6 mice were injected intraperitoneally with PBS or 4 ml/kg CCl_4_ (diluted by olive oil, dilution ratio 1:19). Briefly, mice were administrated with vehicle or CCl_4_ for 3 weeks and were randomized into three groups including vehicle group, CCl_4_ group, and CCl_4_ + CKI group. For CKI treatment, the mice were administered intraperitoneally with CKI (2.5, 5.0, and 7.5 ml/kg) daily for 3 or 6 weeks along with CCl_4_ administration.

To establish MCD diet‐induced NASH model, C57BL/6 mice were fed with normal or MCD diet. Briefly, mice were treated with normal diet or MCD diet for 2 weeks and were randomly divided into vehicle, MCD, and MCD + CKI group. For CKI treatment, CKI (7.5 ml/kg) was administered intraperitoneally for 2 weeks along with MCD diet.

To establish CCl_4_‐induced HCC models, C57BL/6 mice were injected intraperitoneally with 4 ml/kg CCl_4_ (diluted by olive oil, dilution ratio 1:19) three times per week for 25 weeks. Briefly, mice were randomized into three groups including vehicle, CCl_4_, and CCl_4_ + CKI group. For CKI treatment, the mice were administered intraperitoneally with CKI (7.5 ml/kg) daily started on 15th week for 10 weeks along with CCl_4_ administration.

All mice were sacrificed at indicated time points. Mouse liver tissues and serums were harvested for experiments.

### 2.3 Biochemical parameters

The liver function of mice was evaluated by the levels of aspartate transaminase (AST), alanine transaminase (ALT), and total bilirubin (T‐BIL) in mouse serum. The contents of ALT, AST, and T‐BIL were tested by using standard autoanalyzer methods on Chemray 240 automatic biochemistry analyzer (Rayto, USA).

### 2.4 Histology

First, we used 4% paraformaldehyde solution (Sangon Biotech, Shanghai, China) to fix mouse liver tissues. Then, liver tissues were embedded by paraffin. All tissue slices were stained with hematoxylin and eosin (H&E) for morphologic analysis. The determination of collagen deposition was accomplished by Sirius Red or Masson's trichrome staining according to standard procedures. The liver fibrosis stage was assessed by Ishak scale.^22^

### 2.5 Immunohistochemistry

Immunohistochemistry was conducted with primary antibodies against Collagen I (Abcam, cat# ab34710, 1:200), F4/80 (CST, cat# 70076, 1:250), proliferating cell nuclear antigen (PCNA) (Abcam, cat# ab29, 1:5000), αSMA (Abcam, cat# ab5694, 1:200), desmin (Abcam, cat# ab15200, 1:200), cleaved caspase 3 (CST, cat# 9661, 1:200), AFP (Proteintech, cat# 14550‐1‐AP, 1:50), CK19 (Proteintech, cat# 10712‐1‐AP, 1:500), Smad7 (Proteintech, cat# 25840‐1‐AP, 1:200), TGF‐β receptor type 1 (TGFβR1) (Abcam, cat# 31013, 1:50), and Ki67 (CST, cat# 12202, 1:200). In brief, paraffin‐embedded liver tissues were deparaffinized and rehydrated. After antigen was retrieved, the tissues were stained with primary antibodies overnight at 4°C and HRP‐conjugated secondary antibodies at 37°C for 1 h, and target proteins were visualized with diaminobenzidine staining.

### 2.6 Immunofluorescence

Immunofluorescence was accomplished with primary antibodies against αSMA (Abcam, cat# ab5694, 1:200), αSMA (Sigma, cat# A5228, 1:500), desmin (Abcam, ab15200, 1:200), TGFβR1 (Abcam, cat# 31013, 1:200), p‐Smad2/3 (CST, cat# 8828, 1:200), and Smad7 (Santa Cruz, cat# sc‐365846, 1:50). In brief, paraffin‐embedded liver tissues were deparaffinized and rehydrated. After antigen retrieved tissues were stained with primary antibodies overnight at 4°C, they were subsequently stained with fluorescein‐labeled secondary antibodies (Alexa Fluor 488 Goat anti‐Rabbit IgG, Invitrogen, cat# A11034, 1:500; Alexa Fluor Plus 555 Goat anti‐Mouse IgG, Invitrogen, cat# A32727, 1:500) at 37°C for 1 h. After staining with DAPI (ThermoFisher, cat# D21490, 1:1000), the target proteins were visualized with confocal laser scanning microscope.

### 2.7 RNA‐seq analysis

RNA‐seq analysis was performed according to standard procedures, including RNA quantification and qualification, library preparation for transcriptome sequencing, clustering, and sequencing. Sequencing of total RNA from mice liver tissues after indicated treatments was accomplished by Novogene (Beijing, China). For data analysis, differentially expressed genes were analyzed using the DESeq2 R package. Genes with an adjusted *p*‐value <.05 were recognized as differentially expressed. Gene set enrichment analysis (GSEA) was implemented by the Cluster Profiler R package (www.r‐project.org) to calculate the enrichment score of TGF‐β/Smad pathway embedded in the Molecular Signatures Database based on the mice liver RNA‐sequence data.

### 2.8 Flow cytometry

Fresh mouse liver tissues were harvested, minced, and digested into single cell with mouse liver dissociation kits (Miltenyi Biotech) according to the manufacturer's instructions. Lysis buffer (BD Pharmingen, NJ, USA) was used to remove red blood cells. Fixable Viability Stain 780 (BD Pharmingen) was used to exclude the dead cells. Later, cells were blocked with the anti‐CD16/32 antibody (clone 2.4G2) for 10 min, and then incubated with fluorescently conjugated mAbs against mouse CD45, Ly6C, CD11b, and F4/80 (Biolegend, San Diego, CA) for 30 min at 4°C in the dark. Cells were detected by BD FACS Aria II and analyzed with FlowJo software. Macrophages in the liver tissue were characterized by the gating strategy CD45^+^Ly6C^+^CD11b^+^F4/80^+^.

### 2.9 Quantitative real‐time PCR

First, total RNA was extracted from indicated cells by using Trizol reagent (Invitrogen, San Diego, CA) and reverse‐transcribed into cDNA with PrimeScript kit (Takara, Osaka, Japan). Quantitative real‐time PCR was conducted on 7900HT fast real‐time PCR system (Applied Biosystem) using SYBR green as the detection fluorophore. The expression of target gene was normalized to the housekeeping gene GAPDH, actin, or β‐actin. Relative mRNA expression was quantified by the △△C_t_ method. The primer sequences are provided in Tables S2 and S3.

### 2.10 Western blotting

Mouse liver tissues or cells were lysed in lysis buffer (Beyotime Biotechnology, Shanghai, China). Bicinchoninic acid protein assay (Thermo Fisher) kit was used to quantify the protein concentration. Total protein lysates were separated by gradient gel (10%, PAGE Gel Fast Preparation Kit, Epizyme), transferred to a PVDF membrane, and blotted overnight at 4°C with the primary antibodies (primary antibodies information is provided in Table S4). Blots were rinsed and incubated with HRP‐conjugate secondary antibody.

### 2.11 Immunoprecipitation assay

Cell lysates were incubated with anti‐Smad7 (Santa Cruz, cat# sc‐365846) at 4°C for 6 h, followed by Protein A/G agarose beads (Santa Cruz, cat# sc‐2003) overnight. Beads were washed three times and eluted with SDS loading buffer. Tests for immunoprecipitated proteins were performed by SDS‐PAGE as described before.

### 2.12 siRNA‐mediated Smad7 knockdown

The siRNA targeting human *Smad7* (5′‐GGUUUCUCCAUCAAGGCUUTT‐3′), the siRNA targeting mouse *Smad7* (5′‐GAGGCTGTGTTGCTGTGAA‐3′), and negative control (5′‐UUCUCCGAACGUGUCACGUTT‐3′) were synthesized by Tuoran Biotechnology (Shanghai, China).^23^, ^24^ In brief, 1 × 10^6^ LX‐2 cells were plated into six‐well plate in 2 ml DMEM (high glucose) without FBS and antibiotics 24 h before transfection. Then, cells were transfected with 3 μl of 10 μM siRNA per well by using 9 μl lipofectamine RNAiMAX reagent (Invitrogen) and Opti‐MEM medium. Medium was changed into fresh DMEM (high glucose), which contained 10% FBS 6 h after transfection. subsequently, after 18 h LX‐2 cells were treated and then harvested for indicated experiments. To knockdown the Smad7 expression in vivo, C57BL/6 mice were treated with Smad7‐siRNA (5 mg/kg) or negative control (NC)‐siRNA through retro‐orbital injection of the venous sinus. After 72 h, the mice liver tissues were harvested and assessed for the knockdown efficiency of Smad7‐siRNA in vivo by qRT‐PCR and Western blotting.

### 2.13 Statistical analysis

The data were analyzed by using GraphPad Prism Version 7. The statistical significance of differences was examined with Student's *t*‐test. Flow cytometry data were analyzed by FlowJo.10. All data are presented as means ± SEM. The *p*‐values < 0.05 at two sides were considered statistically significant.

## 3 RESULTS

### 3.1 CKI relieved liver fibrosis or cirrhosis among hepatitis patients in clinical RCTs

First, we conducted a meta‐analysis to assess the CKI intervention for patients with viral hepatitis‐induced fibrosis or cirrhosis. Total of 983 publications were identified after searching through the electronic database. Of them, a total of 18 trials that met the inclusion criteria were used for meta‐analysis (Figure S2). Overall, there were a total of 1575 patients with hepatitis B‐ or C‐induced fibrosis or cirrhosis in the 18 trials. Among these, there were 790 subjects from intervention group treated with CKI combined with antiviral drug or traditional hepatinica, whereas 785 patients in the control group received only antiviral drug or traditional hepatinica (Tables S5 and S6). All RCTs were conducted in China and the articles were published from 2004 to 2019, with characteristics listed in Table S7. The intervention effects were evaluated by the difference in liver function (AST, ALT, and T‐BIL) and chronic fibrosis‐related indexes (laminin [LN], hyaluronic acid [HA], procollagen type III [COL3], and collagen type IV [COL4]) between intervention group and control group. The serum levels of ALT, AST, and T‐BIL in the intervention group were decreased (*p*‐values < 0.0001) compared to the control group (Figure S3A–C). CKI treatment also notably suppressed the levels of LN, HA, COL3, and COL4 (*p*‐values < 0.0001) (Figure S4A–D). No significant publication bias from RCTs was detected in assessing the protective roles of CKI on liver functions or fibrosis indexes except for the LN (Table S8). Therefore, we speculated that CKI could be an effective treatment agent for chronic liver fibrosis and cirrhosis.

### 3.2 CKI attenuated chronic liver fibrosis of CCl_4_‐ or MCD diet‐treated mice

**FIGURE 1:**
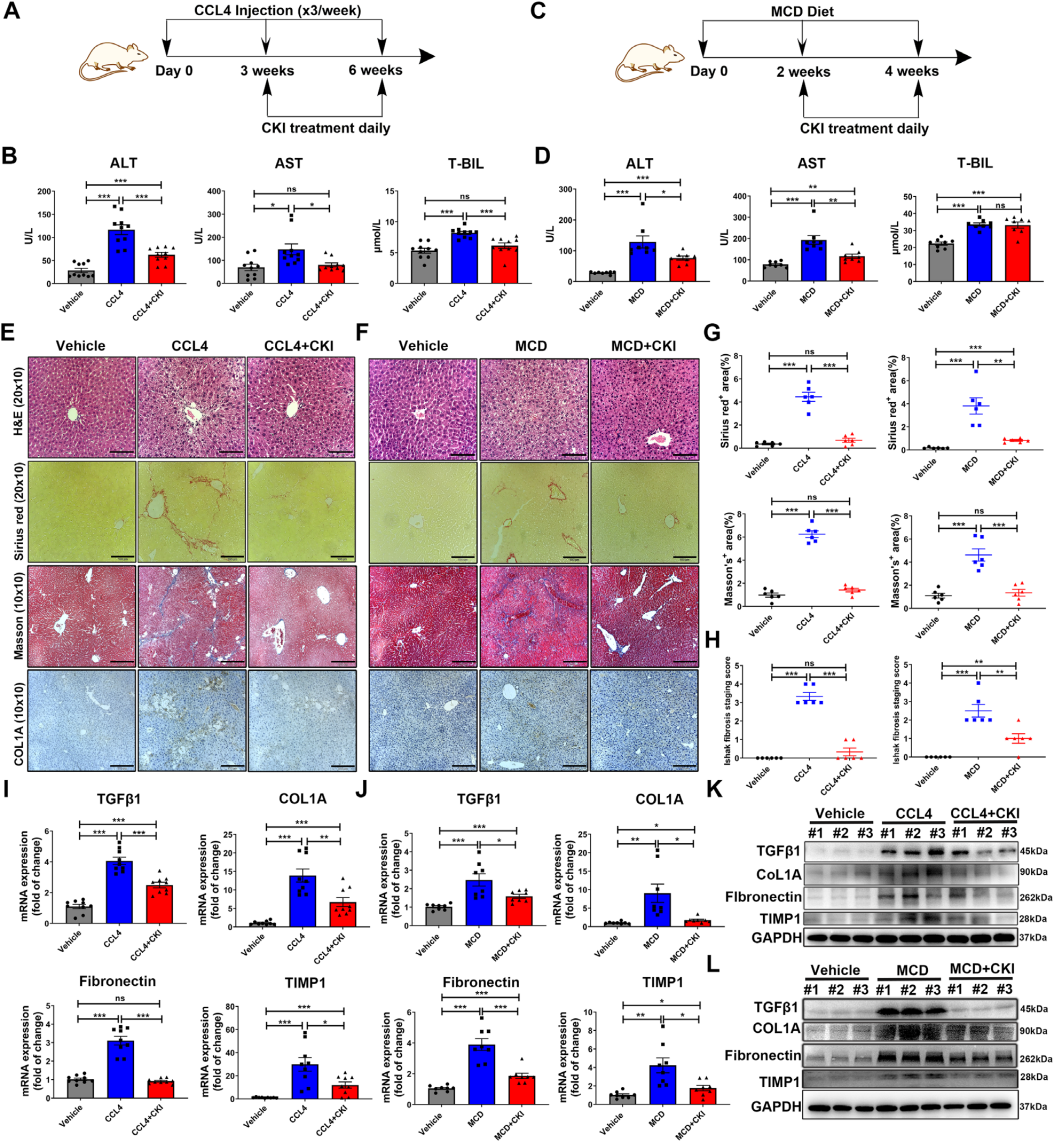
CKI attenuates chronic liver fibrosis. (A) Scheme of experimental procedure for C57BL/6 mice intraperitoneally treated with 4 ml/kg CCl_4_ in olive oil for 6 weeks. Mice were intraperitoneally administrated with CKI (7.5 ml/kg) for 3 weeks, starting at 3 weeks post initiation of CCl_4_ challenge. (B) Serum levels of ALT, AST, and T‐BIL were detected after the final CKI treatment in CCl_4_‐treated mice (*n* = 10). (C) Scheme of experimental procedure for C57BL/6 mice fed with MCD diet for 4 weeks. Mice were intraperitoneally administrated with CKI (7.5 ml/kg) for 2 weeks, starting at 2 weeks post initiation of MCD diet challenge. (D) Serum levels of ALT, AST, and T‐BIL were quantified after the final CKI treatment in MCD diet‐treated mice (*n* = 8). (E and F) Mice liver sections from CCl_4_‐induced or MCD diet‐induced liver fibrosis models were collected for H&E (original magnification 20 × 10, scale bar 110 μm), Sirius Red (original magnification 20 × 10, scale bar 100 μm), Masson staining (original magnification 10 × 10, scale bar 220 μm), and collagen type1 (COL1A, original magnification 10 × 10, scale bar 210 μm) immunostaining after the final CKI treatment (*n* = 6). (G) Positive Sirius Red (above) or Masson staining (below) area were quantified by ImageJ analysis (*n* = 6). (H) Ishak fibrosis score of the Sirius Red‐stained liver sections (*n* = 6). (I and J) mRNA expression of *TGF‐β1*, *COL1A*, *Fibronectin*, and *TIMP1* were analyzed by qRT‐PCR in liver tissues from CCl_4_‐challenged (*n* = 9) or MCD diet‐challenged (*n* = 8) mice. (K and L) Western blot assay for detecting the expression of TGF‐β1, COL1A, Fibronectin, and TIMP1 in liver tissues from CCl_4_‐challenged (K) or MCD diet‐challenged (L) mice. Data are presented as means ± SEM. ns, *p *> 0.05; *p < 0.05; **p < 0.01; ***p < 0.001

To further explore the efficacy of CKI on liver fibrosis and related mechanism, we established CCl_4_‐induced chronic liver fibrosis models (Figure 1A) and MCD diet‐induced NASH model (Figure 1C). Mice were ip treated daily with CKI (7.5 ml/kg) or vehicle for a short‐term (2‐3 weeks, Figure 1) or a long‐term period (6 weeks, Figure S5). After the final CKI treatment, the mouse liver tissues and serum samples were harvested. The mouse serum levels of ALT, AST, and T‐BIL and liver histological examination were measured to evaluate the effect of CKI on liver function and liver fibrosis. We found that the serum levels of ALT (CCl_4_, *p*‐value < 0.0001; MCD, *p*‐value = 0.0001), AST (CCl_4_, *p*‐value = 0.0107; MCD, *p*‐value < 0.0001), and T‐BIL (CCl_4_, *p*‐value < 0.0001; MCD, *p*‐value < 0.0001) were significantly increased in CCl_4_‐ (Figure 1B) or MCD diet‐(Figure 1D) challenged mice, indicating severe liver damage compared to vehicle group. Meanwhile, long‐term CCl_4_ exposure accelerated the severity of liver damage (Figure S5B). Moreover, CCl_4_ or MCD diet treatment induced the liver fibrosis based on liver histological analysis using H&E, Sirius Red staining, Masson staining, and collagen type I (COL1A) immunohistochemistry (Figure 1E–H and Figure S5C–E). Of note, the Sirius Red, Masson staining, and immunostaining of COL1A revealed a significant increase of collagen deposition in mice livers after CCl_4_ or MCD diet treatment (Figure 1E–H and Figure S5C–E).

In contrast, CKI treatment resulted in a decrease in serum levels of ALT (CCl_4_, *p*‐value = 0.0002; MCD, *p*‐value = 0.0231) and AST (CCl_4_, *p*‐value = 0.0158; MCD, *p*‐value = 0.0047) in CCl_4_ or MCD diet‐treated mice (Figure 1B,D and Figure S5B). CKI treatment also reduced the serum levels of T‐BIL in CCl_4_‐treated mice (*p*‐value = 0.0007), whereas had no effect in MCD diet‐treated mice (*p*‐value = ​0.8668) (Figure 1B,D and Figure S5B). Importantly, CKI treatment mitigated liver fibrosis based on the improved liver histological features and decreased collagen deposition in CCl_4_‐ or MCD diet‐treated mice (Figure 1E–H and Figure S5C–E). At the same time, we assessed whether there was any dose‐dependent effect of CKI against liver fibrosis. The therapeutic effect of different dosages of CKI treatment (2.5, 5.0, and 7.5 ml/kg) were evaluated in CCl_4_‐induced model (Figure S6A). The results showed that CKI improved mice liver function and liver histological features, and inhibited collagen deposition in a dose‐dependent manner (Figure S6B–E). Moreover, we also assessed the influence of CKI treatment on normal mice (Figure S7A–D). Compared to vehicle group, CKI treatment had no effect on the liver function, liver histological features, and liver collagen deposition of normal mice (Figure S7A–C). No bodyweight loss was observed during CKI treatment (Figure S7D–G), suggesting that CKI did not have significant host toxicity at these effective doses. In line with histological analysis, mRNA and protein expression of pro‐fibrotic genes (e.g., *TGF‐β1*, *COL1A*, *Fibronectin*, and *TIMP1*), which were upregulated by CCl_4_ or MCD diet treatment, were notably decreased in CKI intervention group (Figure 1I–L, Figures S5F and S8). Collectively, these results indicated that CKI was effectively protective against hepatic fibrosis and hepatocellular injury.

### 3.3 CKI ameliorated inflammatory response, oxidative stress, liver compensatory proliferation, and hepatocellular death of mice during chronic fibrosis

**FIGURE 2:**
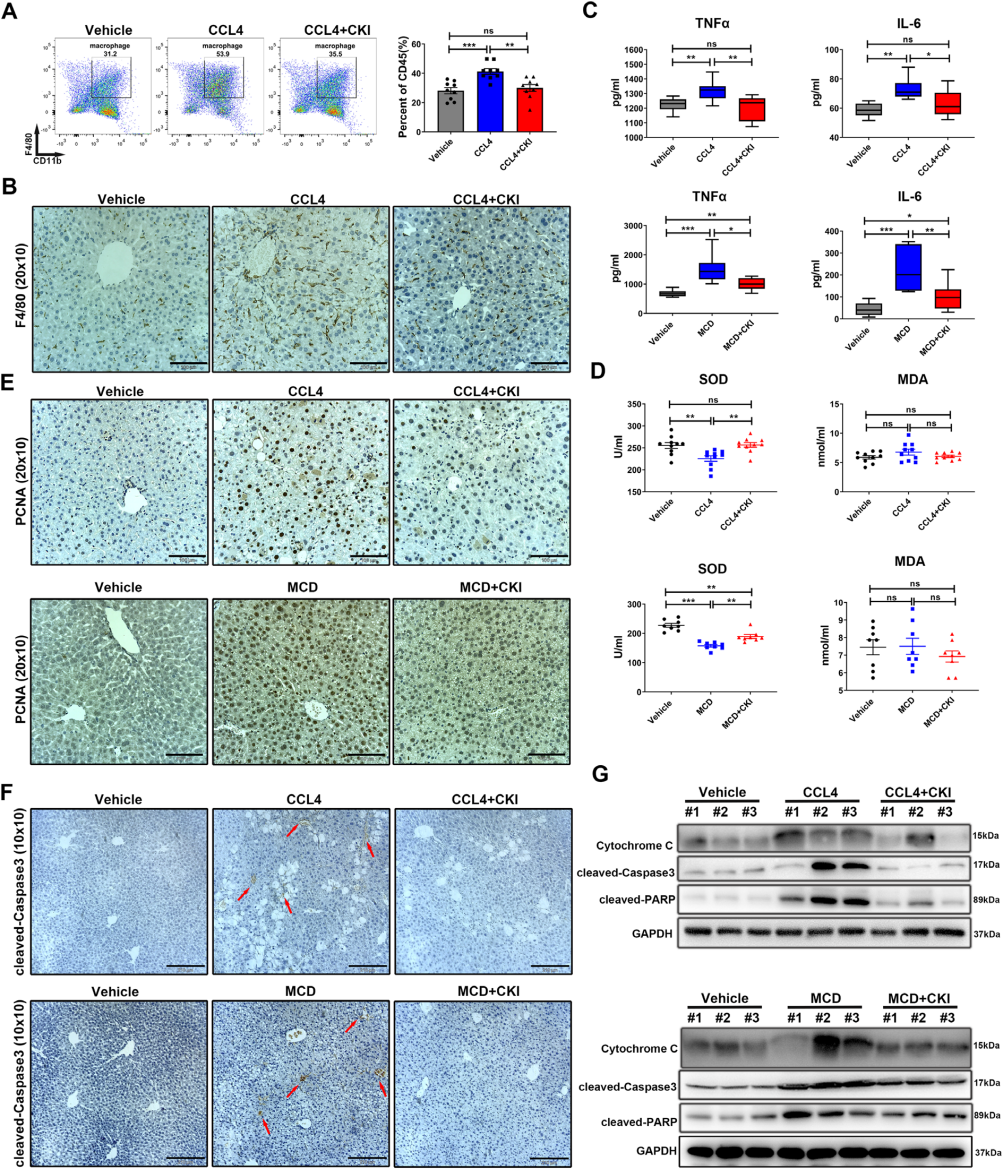
CKI ameliorates inflammatory response, oxidative stress, cell compensatory proliferation, and hepatocellular death in the mice liver. (A) Mice were intraperitoneally treated with 4 ml/kg CCl_4_ for 6 weeks along with CKI treatment (7.5 ml/kg) for 3 weeks. The proportion of macrophages in mice liver tissues was detected by flow cytometry after indicated treatments (right). Representative flow cytometry gating images are shown (left). (B) Representative F4/80 immunostaining of macrophages in mice liver sections were displayed (original magnification 20 × 10, scale bar 100 μm). (C) Serum levels of TNFα and IL‐6 were detected by Elisa assays in CCl_4_‐induced (*n* = 9) or MCD diet‐induced (*n* = 8) liver fibrosis models. (D) Serum levels of superoxide dismutase (SOD) and malondialdehyde (MDA) were quantified by Elisa assays in CCl_4_‐challenged (*n* = 10) or MCD diet‐challenged (*n* = 8) mice. (E) Representative immunostaining of PCNA in the liver section of mice are shown after indicated treatments (original magnification 20 × 10, scale bar 100 μm). (F) Representative immunostaining of cleaved caspase 3 in the mice liver tissues are shown after indicated treatments (original magnification 10 × 10, scale bar 210 μm). (G) Expression of cytochrome C, cleaved caspase 3, and cleaved PARP in liver tissue lysates were determined by Western blot from CCl_4_‐challenged (above) or MCD diet‐challenged (below) mice. Data are presented as means ± SEM. ns, *p *> 0.05; *p < 0.05; **p < 0.01; ***p < 0.001

Liver damage‐induced inflammation is a typical feature of progressive fibrogenesis.^25^ Liver resident macrophages and recruited macrophages promote hepatic inflammation in a tumor necrosis factor α (TNFα)‐dependent manner, leading to liver fibrogenesis.^25^ Flow cytometry of detecting macrophage infiltration or immunostaining of F4/80 revealed that CKI markedly diminished the macrophages infiltration into CCl_4_‐treated mouse livers (Figure 2A,B and Figure S9B). Moreover, CKI was also protective against CCl_4_‐ or MCD diet‐induced severe inflammation as evidenced by decreased serum levels of TNFα and IL‐6 (Figure 2C and Figure S9A). Oxidative stress is highly related to all fibrogenic disorders characterized by chronic tissue damage.^2^ The effect of CKI against oxidative stress was evaluated with the serum superoxide dismutase (SOD) and malondialdehyde (MDA). CKI treatment rescued the serum level of SOD compared to CCl_4_ or MCD diet challenge, while no difference of MDA was observed among these groups (Figure 2D and Figure S9C).

Owing to high regenerative capacity of liver, mice with liver fibrosis have massive hepatocyte death.^4^ We further assessed whether CKI influenced the hepatocyte compensatory response and apoptosis. Immunostaining of PCNA displayed higher numbers of proliferating hepatocytes after CCl_4_ or MCD diet exposure, and CKI intervention reduced the expression of PCNA (Figure 2E and Figure S9D), indicating CKI treatment attenuated CCl_4_‐ or MCD diet‐induced liver compensatory proliferation. Further, CKI reversed the hepatocyte apoptosis as evidenced by the decreased expression of cleaved caspase 3 in the liver immunostaining (Figure 2F). Similarly, Western blotting suggested decreased expression of cytochrome C, cleaved caspase 3, and cleaved PARP in CKI‐treated fibrotic liver tissue (Figure 2G and Figure S10).

### 3.4 CKI reduced liver fibrosis by suppressing HSCs activation in vivo

**FIGURE 3:**
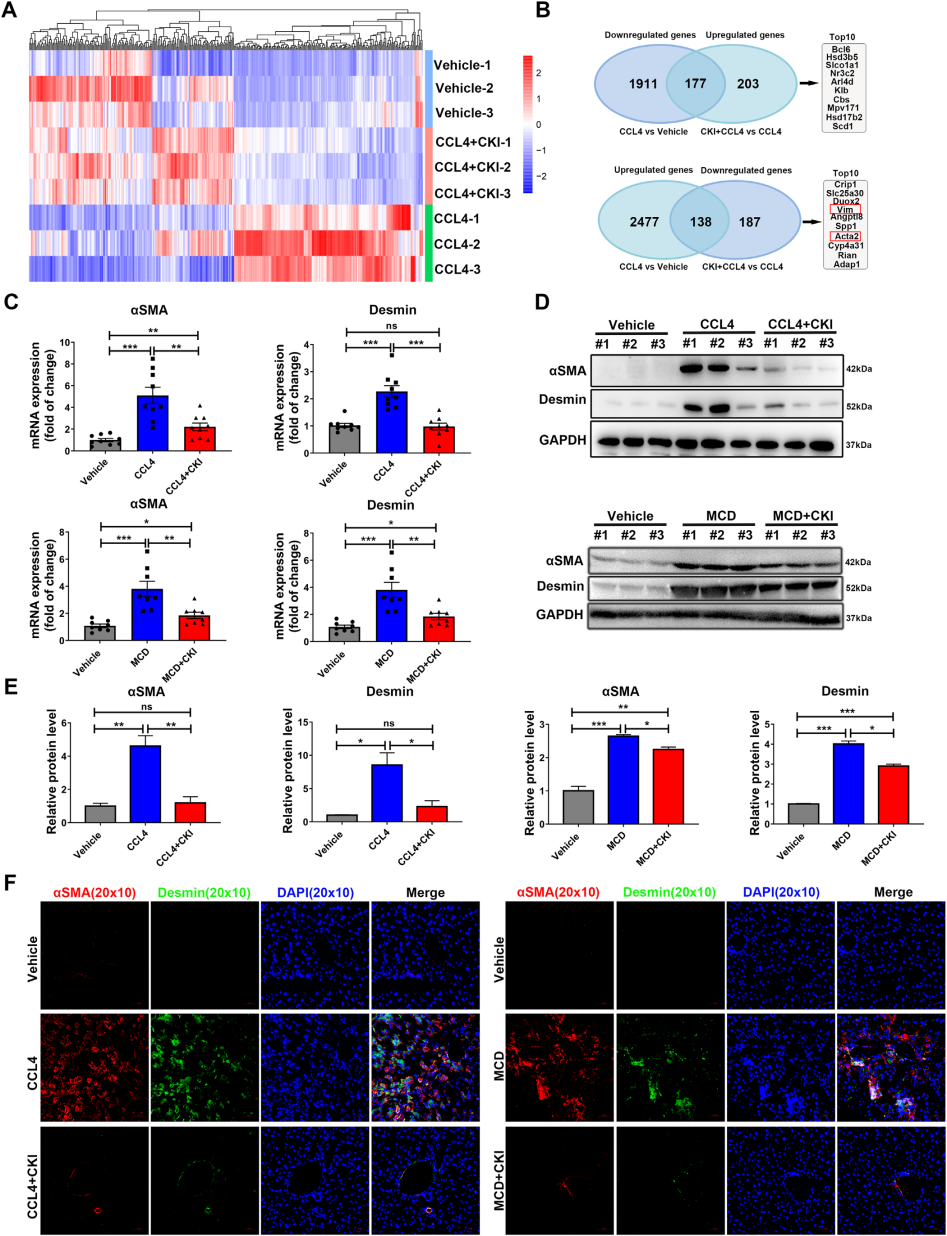
CKI inhibits liver fibrosis by suppressing HSCs activation in vivo. (A) Mice were intraperitoneally treated with 4 ml/kg CCl_4_ for 6 weeks along with CKI treatment (7.5 ml/kg) for 3 weeks. Later, liver tissues were collected for RNA‐sequence (*n* = 3 for each group). Heat map for differentially expressed genes between vehicle, CCl_4_, and CCl_4_ + CKI group. (B) Venn analysis of upregulated genes and downregulated genes from CCl_4_ + CKI versus CCl_4_ treatment and CCl_4_ versus vehicle treatment. The top 10 upregulated genes and downregulated genes are shown from CCl_4_ + CKI versus CCl_4_ treatment. (C) mRNA expression of *αSMA* and *desmin* were analyzed by qRT‐PCR in the liver tissues from CCl_4_‐challenged (above) or MCD diet‐challenged (below) mice. (D) Western blot assay for detecting the expression of αSMA and desmin in mice liver tissues. (E) Quantitative analysis of the protein expression of αSMA and desmin. (F) Representative immunofluorescence staining images of αSMA and desmin of liver sections from CCl_4_‐treated (left) or MCD diet‐treated (right) mice (original magnification 20 × 10, scale bar 50 μm). Data are presented as means ± SEM. ns, *p *> 0.05; *p < 0.05; **p < 0.01; ***p < 0.001

To identify the mechanism of CKI's antifibrosis effect, we first explored whether CKI interfered with the CCl_4_ metabolism. CCl_4_ is metabolized by cytochrome P450 subfamily 2E1 (CYP2E1) to the highly reactive trichloromethyl free radical, which causes hepatocellular damage through lipid peroxidation. Expression of CYP2E1 was downregulated in liver tissues after CCl_4_ injury (Figure S11), reportedly due to phosphorylation‐dependent protein degradation.^26^ Compared to only CCl_4_ treatment, combinative administration of CKI and CCl_4_ exhibited the similar effect on CYP2E1 expression, indicating CKI did not affect CCl_4_ metabolism in vivo (Figure S11). We further performed a deep RNA‐seq analysis of mouse liver tissues from healthy and CCl_4_‐challenged mice with or without CKI intervention. Compared to vehicle group, CCl_4_ challenge showed a rather distinct gene profile, whereas CKI treatment alleviated aberrantly expressed genes expression induced by CCl_4_ challenge (Figure 3A). In total, the expression of 315 genes was modified by CKI intervention in the CCl_4_‐treated mice. Specifically, 1911 genes were downregulated in CCl_4_ treatment group, whereas CKI treatment upregulated 177 genes expression; 2477 genes were upregulated in CCl_4_ treatment group, whereas CKI treatment downregulated 138 genes expression (Figure 3B).

HSCs have a critical role in inducing liver fibrosis, which lead to increased extracellular matrix production and decreased degradation and have been considered as a potential therapeutic target in liver fibrosis.^27^ We found CKI treatment dramatically repressing the expression of HSCs activation markers—αSMA (*Acta2*) and Vimentin (*Vim*), which were among the top 10 CKI rescued genes (Figure 3B). The upregulation of activated HSC markers αSMA and desmin in both mRNA and protein level were also confirmed in CCl_4_‐treated or MCD diet‐treated mouse livers, but CKI significantly suppressed their expressions (Figure 3C–E and Figure S12A–C). In accordance, staining of αSMA and desmin in liver tissues further confirmed the obvious effect of CKI on reducing αSMA and desmin positive area (Figure 3F and Figure S12D). Therefore, our results revealed that CKI might reduce liver fibrosis by suppressing HSCs activation.

### 3.5 CKI inhibited TGF‐β/Smad signaling in mice liver

**FIGURE 4:**
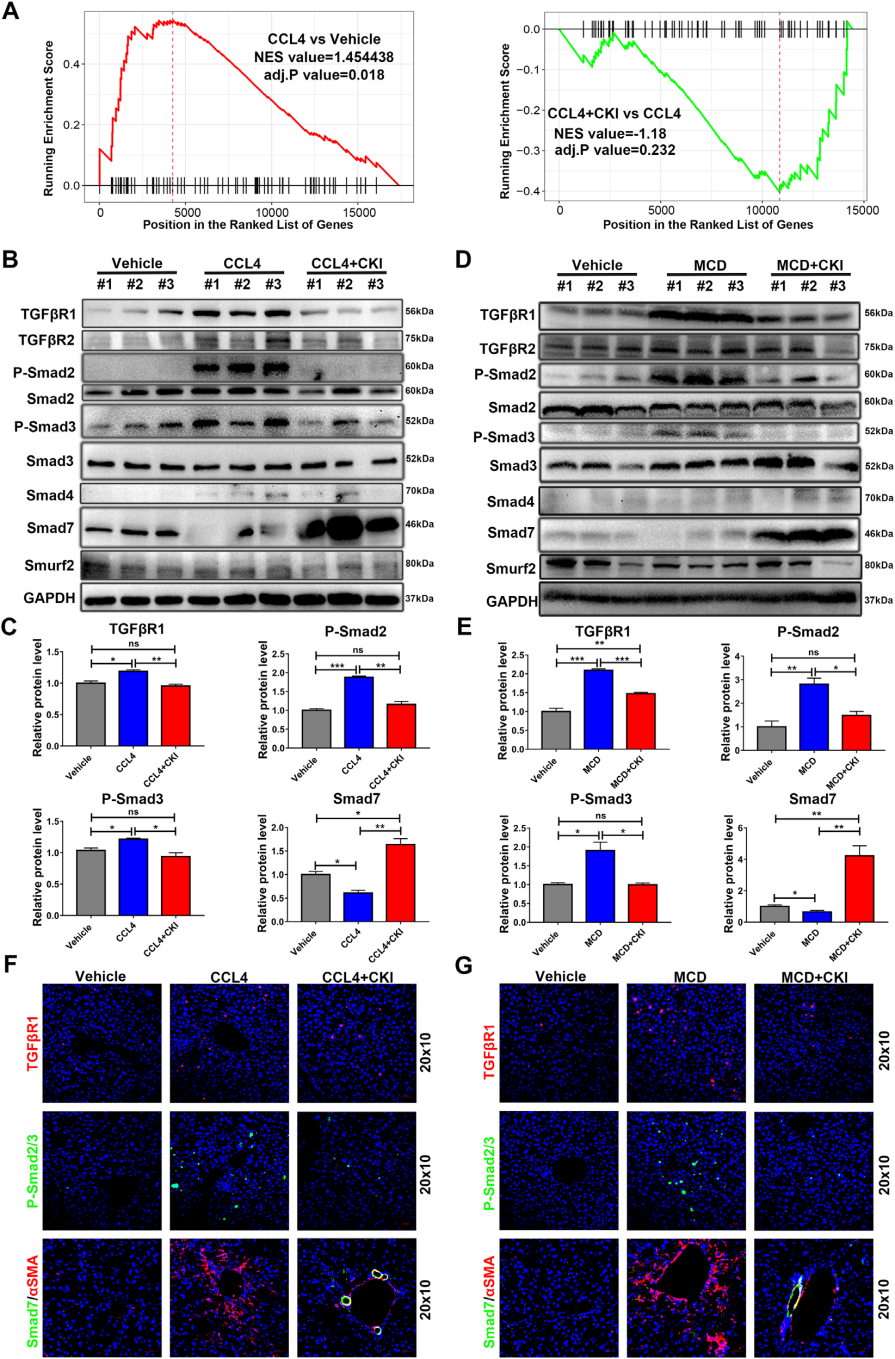
CKI inhibits TGF‐β/Smad signaling in hepatic stellate cells. (A) GSEA for TGF‐β pathway enrichment score in CCl_4_ versus vehicle group (left) and CCl_4_ + CKI versus CCl_4_ (right) group. (B and D) Western blot analysis of TGFβR1, TGFβR2, p‐Smad2, total Smad2, p‐Smad3, total Smad3, Smad4, Smad7, and Smurf2 in liver tissue lysates from CCl_4_‐treated or MCD diet‐treated mice. (C and E) Quantitative analysis of protein expression of TGFβR1, p‐Smad2, p‐Smad3, and Smad7. (F and G) Representative immunofluorescence staining images of TGFβR1, p‐Smad2/3, and Smad7 of liver sections from CCl_4_‐treated or MCD diet‐treated mice (original magnification 20 × 10, scale bar 50 μm). Data are presented as means ± SEM. ns, *p *> 0.05; *p < 0.05; **p < 0.01; ***p < 0.001

Previous study has suggested a crucial role of TGF‐β/Smad signaling in activating HSCs and chronic fibrosis in the liver.^28^ We evaluated the activation of TGF‐β/Smad axis in mouse livers after CCl_4_ or MCD diet challenge. GSEA for the RNA‐seq data suggested that CCl_4_ challenge apparently activated TGF‐β pathway in mouse livers. However, it was reversed following CKI treatment (Figure 4A). Analysis of hepatic expression of TGF‐β/Smad signaling‐related proteins demonstrated that CCl_4_‐ or MCD diet‐treated liver exhibited increased expression of TGFβR1, p‐Smad2, and p‐Smad3 and decreased expression of Smad7, which were further reversed by CKI treatment (Figure 4B–E). The expression of TGF‐β receptor type 2 (TGFβR2), Smad2, Smad3, Smad4, and Smurf2 was not altered among these groups (Figure 4B,D and Figure S13). Similarly, immunofluorescence staining of TGFβR1, p‐Smad2/3, and Smad7 confirmed these findings (Figure 4F,G and Figure S14). Of note, Smad7 was colocalized with αSMA, suggesting that CKI selectively induced Smad7 expression in activated HSCs (Figure 4F,G and Figure S14). Collectively, these results suggested that CKI inhibited TGF‐β/Smad signaling during chronic fibrosis.

### 3.6 CKI selectively suppressed HSCs activation by restraining TGF‐β/Smad signaling

**FIGURE 5:**
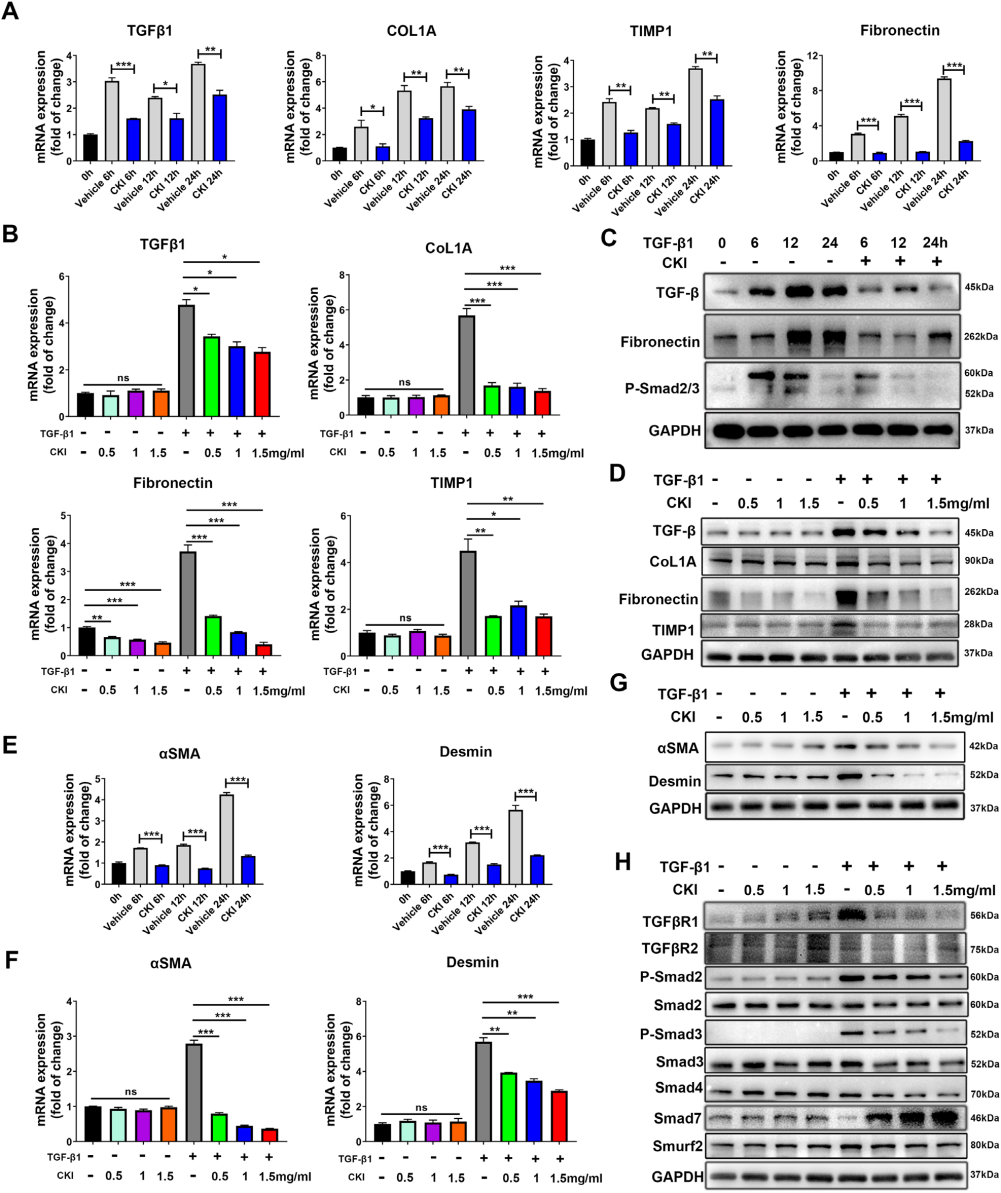
CKI suppresses HSCs activation by restraining TGF‐β/Smad signaling in vitro. (A) LX‐2 cells were treated with 5 ng/ml TGF‐β1 along with 1 mg/ml CKI for 6, 12, and 24 h. mRNA expressions of *TGF‐β1*, *COL1A*, *Fibronectin*, and *TIMP1* were detected by qRT‐PCR in LX‐2 cell lysates. (B) LX‐2 cells were treated with or without 5 ng/ml TGF‐β1 along with different dosages of CKI (0.5, 1, and 1.5 mg/ml) for 12 h. mRNA expressions of *TGF‐β1*, *COL1A*, *Fibronectin*, and *TIMP1* were detected by qRT‐PCR in LX‐2 cell lysates. (C) Western blot for TGFβ, Fibronectin, and p‐Smad2/3 in LX‐2 cells from A. (D) Western blot for TGFβ, COL1A, Fibronectin, and TIMP1 protein levels in LX‐2 cells from B. (E and F) qRT‐PCR analysis of HSCs activation markers *αSMA* and *desmin* mRNA expression in LX‐2 cells. (G) Protein expressions of αSMA and desmin were quantified by Western blot in LX‐2 cells. (H) Western blot analysis of TGFβR1, TGFβR2, p‐Smad2, total Smad2, p‐Smad3, total Smad3, Smad4, Smad7, and Smurf2 in LX‐2 cells. Data are presented as means ± SEM. ns, *p *> 0.05; *p < 0.05; **p < 0.01; ***p < 0.001

We postulated that CKI may exert the antihepatic fibrosis effect by suppressing HSCs activation through restricting TGF‐β/Smad signaling pathway. To test whether CKI inhibited HSC trans‐differentiation, we employed in vitro Hepatic stellate cell LX‐2 modeling. We assessed the influence of different dosages of CKI (0.5, 1, and 1.5 mg/ml) on TGF‐β1‐induced LX‐2 trans‐differentiation at different time intervals (6, 12, and 24 h) (Figure 5). RNA (Figure 5A,B) and protein‐based (Figure 5C,D and Figure S15A,B) analyses confirmed that CKI resulted in diminished expression of liver fibrosis‐related genes *TGF‐β1*, *COL1A*, *Fibronectin*, and *TIMP1* in a dose‐dependent manner, which were upregulated in TGF‐β1‐treated LX‐2 cells. Further, CKI was also protective against HSCs activation as evidenced by impairment of TGF‐β1‐induced αSMA and desmin expression (Figure 5E–G and Figure S15C). Of note, CKI had no effect on the expression of TGF‐β1, COL1A, TIMP1, αSMA, and desmin in quiescent HSC cells (Figure 5B,D,F,G and Figure S15B,C) or in cultured hepatocyte cells with or without TGF‐β1 treatment (Figure S16A–F). These results suggested that CKI selectively inhibited TGF‐β1‐induced HSCs activation. Next, we explored how CKI regulated the TGF‐β/Smad signaling in HSCs activation. Western blotting suggested that ‐p‐Smad2, p‐Smad3, and TGFβR1 were significantly increased, whereas Smad7 was markedly decreased after TGF‐β treatment in cultured HSCs, which were effectively blocked by CKI treatment (Figure 5H and Figure S15D). CKI had no effect on the expression of TGFβR2, Smad2, Smad3, Smad4, and Smurf2 (Figure 5H and Figure S15E). Interestingly, CKI had a marginal effect on TGF‐β‐induced changes of p‐Smad2, p‐Smad3, and Smad7 in cultured hepatocyte cells (Figure S16G,H).

### 3.7 Oxymatrine and sophocarpine were identified as the most antifibrotic ingredients in CKI

To identify the material basis responsible for the antifibrosis effect of CKI, we further explored the influence of four identified alkaloids and Mimic on the activation and fibrosis of LX‐2 cells (Table S1), including matrine (M), oxymatrine (OM), sophocarpine (S), oxysophocarpine (OS) and Mimic (M+OM+S+OS). We found that matrine, oxymatrine, sophocarpine, and oxysophocarpine partly suppressed the mRNA and protein liver fibrosis‐related genes *TGF‐β1*, *COL1A*, *Fibronectin* and *TIMP1* (Figure S17A,B,D). Among them, oxymatrine and sophocarpine were the most effective (Figure S17A,B,D). Moreover, the four alkaloids were also protective against HSCs activation as evidenced by impairment of TGF‐β1‐induced αSMA and desmin expression (Figure S17B–D). Further, the four alkaloids Mimic displayed a similar inhibition effect on the activation and fibrosis of LX‐2 cells compared to CKI (Figure S17A–D). Next, we investigated whether the four alkaloids could inhibit TGF‐β/Smad signaling in HSCs activation. Western blotting assays showed matrine, oxymatrine, sophocarpine, and oxysophocarpine decreased the expression of p‐Smad2, p‐Smad3, and TGFβR1 and increased Smad7 expression (Figure S17E,F). Accordingly, the four alkaloids Mimic also exerted a comparable suppression influence on TGF‐β/Smad axis compared to CKI (Figure S17E,F). Hence, the four alkaloids were identified as the material basis of CKI, and oxymatrine and sophocarpine were identified as the most antifibrotic ingredients in CKI.

### 3.8 Smad7 was responsible for inhibitory effect of CKI on HSCs activation

**FIGURE 6:**
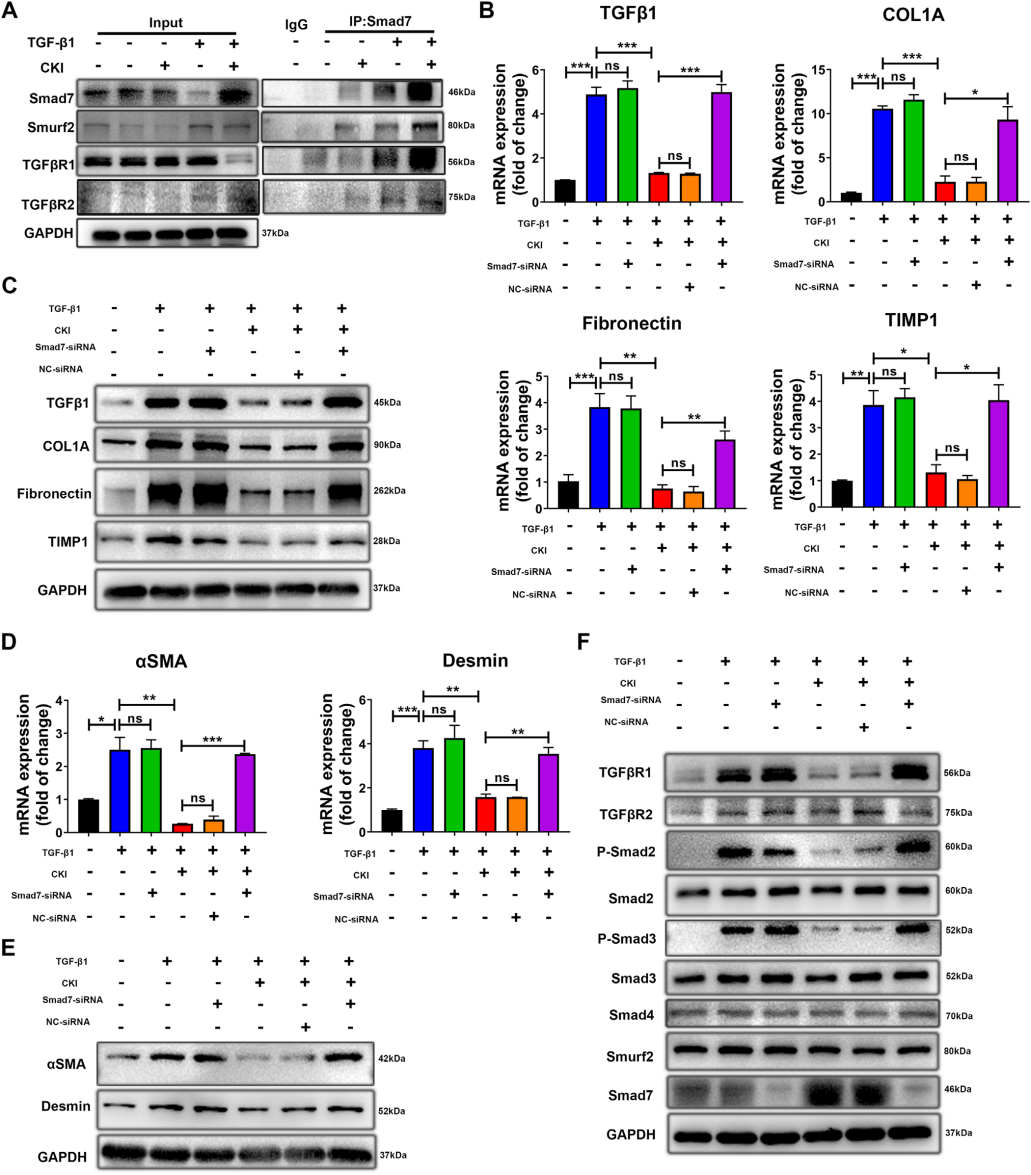
Smad7 is responsible for the inhibitory effect of CKI on HSCs in vitro. (A) LX‐2 cells were treated with or without 5 ng/ml TGF‐β1 along with 1 mg/ml CKI for 12 h, and the binding between Smad7 with Smurf2, TGFβR1, and TGFβR2 was determined by coimmunoprecipitation. (B) Smad7‐knockdown LX‐2 cells were treated with or without 5 ng/ml TGF‐β1 along with 1 mg/ml CKI for 12 h. mRNA expressions of *TGF‐β1*, *COL1A*, *Fibronectin*, and *TIMP1* were detected by qRT‐PCR in Smad7‐knockdown LX‐2 cells. (C) Western blot for TGFβ, COL1A, Fibronectin, and TIMP1 protein levels in Smad7‐knockdown LX‐2 cells. (D) qRT‐PCR analysis of *αSMA* and *desmin* mRNA expression in Smad7‐knockdown LX‐2 cells. (E) Protein expressions of αSMA and desmin were quantified by Western blot in Smad7‐knockdown LX‐2 cells. (F) Western blot analysis of TGFβR1, TGFβR2, p‐Smad2, total Smad2, p‐Smad3, total Smad3, Smad4, Smad7, and Smurf2 in Smad7‐knockdown LX‐2 cells. Data are presented as means ± SEM. ns, *p *> 0.05; *p < 0.05; **p < 0.01; ***p < 0.001

Smad7 has been shown to inhibit the activation of HSCs and prevent liver fibrosis.^29^ Since the in vivo and in vitro results showed that CKI selectively upregulated Smad7 expression in activated HSCs (Figure 4B–G and Figure 5H), we speculated that Smad7 might be a crucial target of CKI for its antifibrosis effect. Smad7 acts as a scaffold to recruit Smurf2 to the TGF‐β receptor complex to facilitate TGF‐β receptor polyubiquitination and complex degradation.^30^, ^31^ We further explored the effect of CKI on the interaction in the Smad7, Smurf2, TGFβR1, and TGFβR2 protein complex in the activated HSCs. The coimmunoprecipitation results indicated that the interaction among Smad7, Smurf2, and TGFβR1 were enhanced (Figure 6A), suggesting CKI facilitated TGFβR1 degradation via formation of Smad7‐mediated protein complex.

We next investigated whether Smad7 was required for CKI‐mediated antifibrosis and inactivation of HSCs. SiRNA‐mediated knockdown of Smad7 was conducted in LX‐2 cells (Figure S18A,B). Depletion of Smad7 had no effect on TGF‐β1, COL1A, Fibronectin, TIMP1, αSMA, and desmin expression in quiescent (Figure S18C,D) or TGF‐β1‐activated LX‐2 cells (Figure 6B,C and Figure S19A), but completely abolished the inhibitory effect of CKI on these pro‐fibrosis proteins expression (Figure 6B,C and Figure S19A). Importantly, silencing of Smad7 eventually abrogated CKI‐mediated reduction of the combinational markers involved in HSCs activation including αSMA and desmin (Figure 6D,E and Figure S19B). Consistently, CKI significantly reduced the expression of TGFβR1, p‐Smad2, and p‐Smad3 in TGF‐β1‐activated LX‐2 cells, and the expression of these proteins was almost completely conversed in Smad7‐silenced cells (Figure 6F and Figure S19C), indicating that CKI inhibits HSCs activation and TGF‐β signaling through enhancing Smad7 expression. Together, these data suggested that CKI rebalanced TGF‐β/Smad7 signaling in HSCs, thereby inhibiting chronic liver fibrosis.

### 3.9 CKI attenuated chronic liver fibrosis by targeting Smad7 in HSCs

**FIGURE 7:**
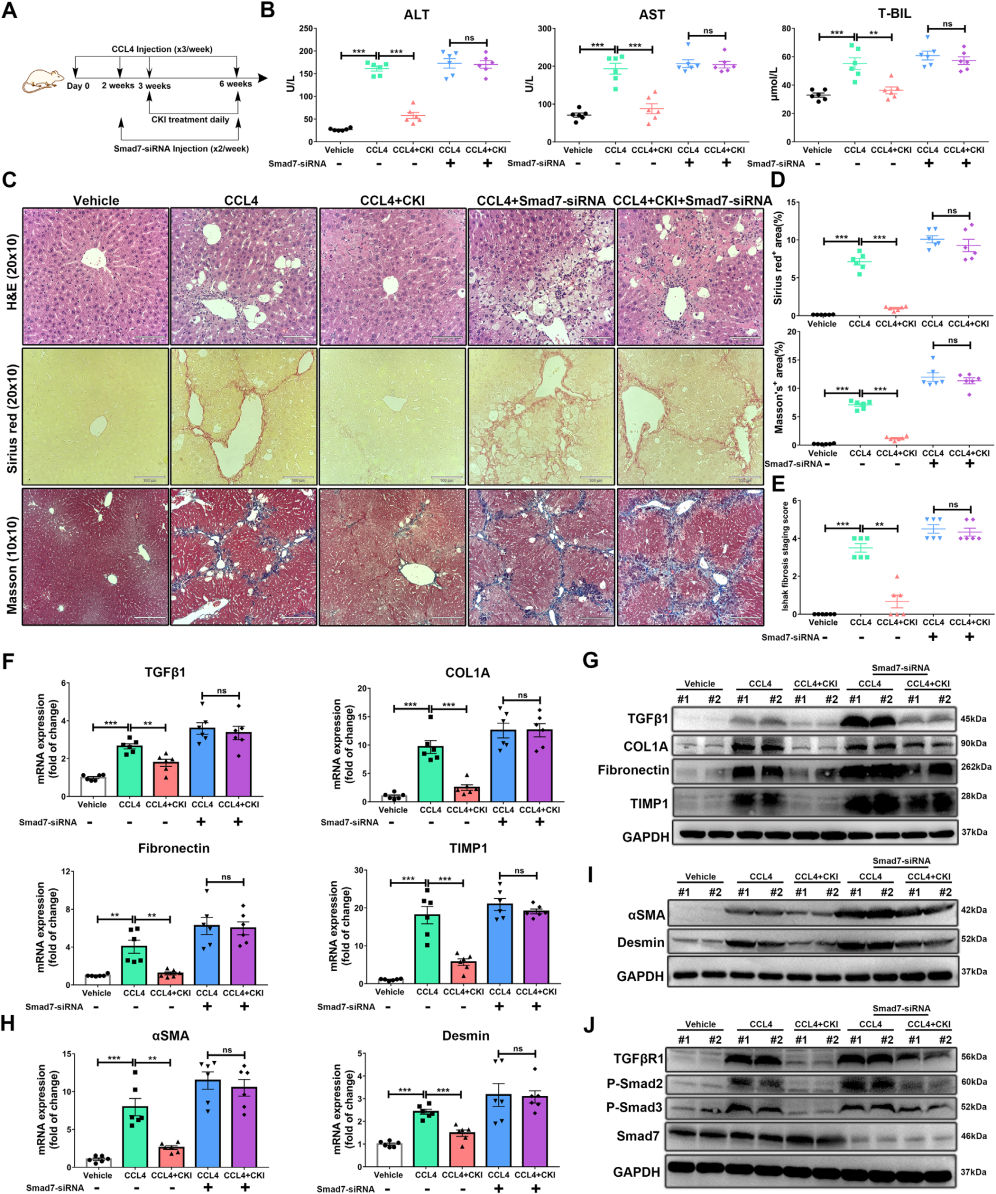
CKI attenuates chronic liver fibrosis through targeting Smad7 in HSCs. (A) Scheme of experimental procedure for C57BL/6 mice intraperitoneally treated with 4 ml/kg CCl_4_ in olive oil for 6 weeks. Mice were intraperitoneally administrated with CKI (7.5 ml/kg) for 3 weeks, starting at 3 weeks post initiation of CCl_4_ challenge. Mice were treated with Smad7‐siRNA (5 mg/kg) through retro‐orbital injection of the venous sinus to knockdown Smad7 expression, starting at 2 weeks post initiation of CCl_4_ challenge. (B) Serum levels of ALT, AST, and T‐BIL were detected in each indicated group (*n* = 6). (C) Mice liver sections from CCl_4_‐induced liver fibrosis models were collected for H&E (original magnification 20 × 10, scale bar 100 μm), Sirius Red (original magnification 20 × 10, scale bar 100 μm), and Masson staining (original magnification 10 × 10, scale bar 210 μm) after the final CKI treatment (*n* = 6). (D) Positive Sirius Red or Masson staining area were quantified by ImageJ analysis (*n* = 6). (E) Ishak fibrosis score of the Sirius Red‐stained liver sections (*n* = 6). (F) mRNA expressions of *TGF‐β1*, *COL1A*, *Fibronectin*, and *TIMP1* were analyzed by qRT‐PCR in mouse liver tissues. (G) Western blot assay for detecting the expression of TGF‐β1, COL1A, Fibronectin, and TIMP1 in mouse liver tissue. (H) mRNA expressions of *αSMA* and *desmin* were analyzed by qRT‐PCR in the liver tissues from CCl_4_‐challenged mice. (I) Western blot assay for detecting the expression of αSMA and desmin in mouse liver tissues. (J) Western blot assay for detecting the expression of TGFβR1, p‐Smad2, p‐Smad3, and Smad7 in mouse liver tissues. Data are presented as means ± SEM. ns, *p *> 0.05; *p < 0.05; **p < 0.01; ***p < 0.001

To further explore the role of Smad7 in mediating the antifibrosis function of CKI in vivo, the Smad7‐siRNA was employed to knockdown the expression of Smad7 in CCl_4_‐induced chronic liver fibrosis model (Figure 7A). First, we detected the Smad7 knockdown efficiency of Smad7‐siRNA. After 5 mg/kg Smad7‐siRNA injection for 72 h, Smad7‐siRNA effectively knockdown the expression of Smad7 in mouse liver lysates compared to vehicle or NC‐siRNA (Figure S18E,F). We found that Smad7 depletion abolished the therapeutic effect of CKI against chronic liver fibrosis, evidenced by mouse liver function, liver histological features, and collagen deposition (Figure 7B–E). In line with histological analysis, Smad7 depletion also suppressed the inhibitory effect of CKI on the mRNA and protein expression of TGF‐β1, COL1A, Fibronectin, and TIMP1 in mouse livers (Figure 7F,G and Figure S20A). Notably, Smad7 depletion abrogated CKI‐mediated suppression of HSCs activation by reversing the expression of αSMA and desmin (Figure 7H,I and Figure S20B). Accordingly, CKI no longer showed influence on the expression of TGFβR1, p‐Smad2, p‐Smad3, and Smad7 in mouse livers after knocking down Smad7 (Figure 7J and Figure S20C). Taking above, CKI attenuated chronic liver fibrosis by targeting Smad7 in HSCs.

### 3.10 CKI was protective against HCC progressed from fibrosis

**FIGURE 8:**
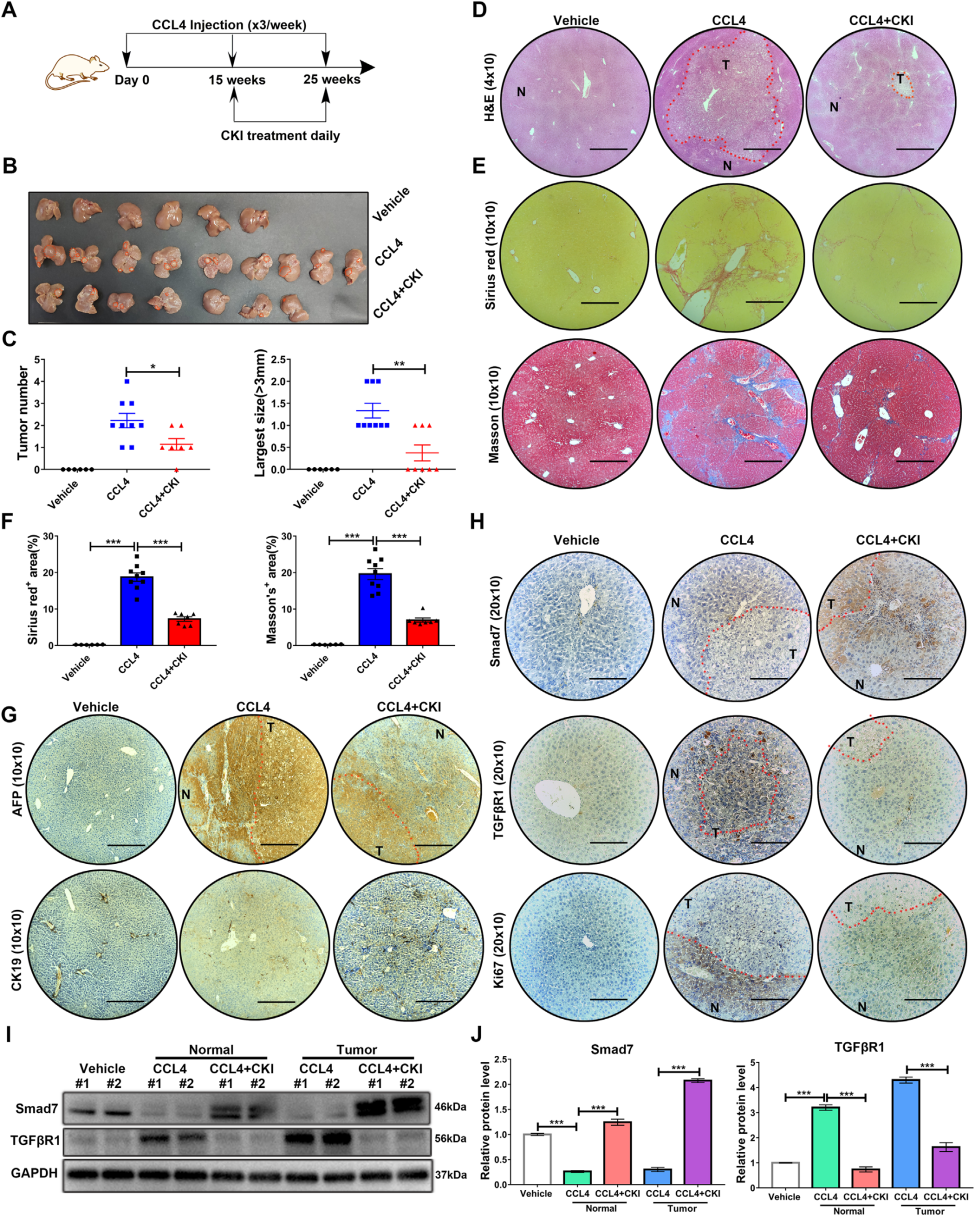
CKI intervention postpones HCC in CCl_4_‐challenged mice. (A) Scheme of experimental procedure for C57BL/6 mice intraperitoneally treated with 4 ml/kg CCl_4_ in olive oil for 25 weeks. Mice were intraperitoneally administrated with CKI at 7.5 ml/kg for 10 weeks, starting at 15 weeks post initiation of CCl_4_ challenge. (B) Photographs of mouse livers from vehicle (*n* = 6), CCl_4_ (*n* = 9), and CKI intervention (*n* = 7) groups. (C) Tumor number and the largest size (>3 mm) were quantified in different groups. (D) Representative images of H&E staining of mouse liver tissues are shown (original magnification 4 × 10, scale bar 890 μm). (E) Representative images of Sirius Red and Masson's trichrome staining are displayed in different treatment groups (original magnification 10 × 10, scale bar 350 μm). (F) Positive Sirius Red or Masson staining area were quantified by ImageJ analysis in each group. (G) Representative immunostaining of AFP and CK19 in mouse liver tissues are shown from indicated treatment (original magnification 10 × 10, scale bar 360 μm). (H) Representative immunostaining of Smad7, TGFβR1, and Ki67 of mice livers are shown (original magnification 20 × 10, scale bar 180 μm). (I) Western blot analysis of Smad7 and TGFβR1 in mice liver tumors and surrounding normal liver tissue. (J) Quantitative analysis of the protein expression of Smad7 and TGFβR1. N, normal liver tissue; T, tumor. Data are presented as means ± SEM. ns, *p *> 0.05; *p < 0.05; **p < 0.01; ***p < 0.001

Since liver fibrosis is the primary risk factor for HCC,^3^ we postulated that CKI would also mitigate malignant transformation in the fibrotic liver. To test this, mice were ip treated with 4 ml/kg CCl_4_ in olive oil for 25 weeks to induce orthotopic HCC, and CKI intervention was started on the 15th week (Figure 8A). Consistent with our hypothesis, CKI protected mice from fibrosis and liver cancer development (Figure 8B–D). CKI treatment reduced the number of tumors and tumor size in the liver of CCl_4_‐treated mice (Figure 8B–D). Moreover, Sirius Red and Masson staining confirmed the decreased collagenous fibers after CKI intervention in normal liver tissues (Figure 8E,F), indicating that CKI suppressed collagen deposition and disrupted progression from chronic fibrosis to HCC. The immunostaining of AFP and CK19 further confirmed that CKI postponed hepatocarcinogenesis (Figure 8G). We further assessed whether CKI treatment had similar effect on Smad7 and TGFβR1 expression between the HCC tumors and its adjacent normal tissues. The immunohistochemistry and Western blotting assays showed an upregulated Smad7 and downregulated TGFβR1 expression in CKI‐treated HCC tumors and its adjacent normal tissues (Figure 8H–J). Moreover, we also performed immunostaining of Ki67 to evaluate cell proliferation. Compared to CCl_4_ group, CKI inhibited abnormal cell proliferation of HCC tumors and surrounding normal liver tissues (Figure 8H), suggesting CKI exerted a similar underlying mechanism between attenuating chronic liver fibrosis and inhibiting HCC formation by upregulating Smad7 expression to inhibit TGF‐β/Smad signaling. Hence, high expression of Smad7 and low expression of TGFβR1 in HCC tumors and surrounding normal liver tissues could be tumor suppressive.

## 4 DISCUSSION

Chronic liver diseases account for about 2 million deaths around the world and are recognized as a major global health burden in China.^32^ Most types of chronic liver diseases cause the extracellular matrix accumulation to destroy the physiological architecture of the liver, finally leading to chronic liver fibrosis.^33^ Currently, the main antifibrotic therapies include hepatocyte protection, inhibition of HSCs activation, extracellular matrix evolution, and immune modulation.^34^, ^35^ Although there are several compounds which have been demonstrated to have antifibrotic activity in the in vitro and in vivo models, none has been thoroughly validated in the clinic or commercialized as a therapy for fibrosis.^7^

Due to the lack of effective therapeutics, there is an urgent need for exploring new antifibrosis treatments. In recent years, drug repurposing is deemed as one of the most effective and fastest strategies in exploring potential drug.^36^ Given previously developed drugs have been explored and examined in terms of pharmacokinetics and biosafety, their development for new uses can be promising, and the development cycle and costs can also be largely reduced.^37^ CKI has been approved by NMPA in China and is being as a TCM formula to treat cancer‐induced pain for over 20 years.^21^ Previous studies found CKI could reduce chemotherapy‐induced toxicity and improve patient's life quality without side effects.^18^, ^38^ Moreover, several RCTs in China also approved the efficacy of CKI in treatment of chronic hepatitis B, chronic hepatitis C, hepatitis liver fibrosis, and hepatitis liver cirrhosis. In this study, we systematically revealed the antifibrosis and anti‐inflammation role of CKI intervention in several murine models with chronic fibrosis. The underlying mechanism of the antifibrotic effect of CKI was also uncovered. Hence, we provide preclinical evidence of a novel application of CKI as a treatment option for chronic fibrosis.

The majority of HCCs occur under the background of clinically significant fibrosis or cirrhosis.^39^ The elimination or appropriate treatment of the underlying etiologies will help to alleviate fibrosis progression and decrease the risk of development of cirrhosis and HCC.^35^ Preventing HBV or HCV infection has been identified as effective in reducing virus‐associated hepatitis or HCC.^40^, ^41^ However, chemoprevention strategies in nonviral‐related HCC remain a significant unmet medical need.^3^ Our results showed that CKI intervention inhibited the tumor size and tumor numbers of HCC, which finally postponed the progression of chronic fibrosis to HCC, suggesting the potential of CKI in clinical use.

Previous researches on CKI were mainly focused on the antitumor effect, including targeting Prdxs/ROS/Trx1 axis to inhibit acute myeloid leukemia,^19^ targeting TRPV1/ERK signaling to suppress sarcoma,^13^ targeting Wnt/β‐catenin pathway to inhibit breast cancer,^17^ and targeting TNFR1‐mediated NF‐κB signaling in tumor‐associated macrophages to remodel HCC microenvironment.^21^ However, there was little research directly focused on the role of Prdxs/ROS/Trx1 or TRPV1/ERK axis in HSCs activation. ROS is produced by multiple liver injuries and promote liver fibrosis by stimulating the production of collagen I in activated HSCs.^42^ However, there are no data related to how upregulated ROS level leads to HSCs activation and trans‐differentiation into myofibroblasts.^9^ In response to various fibrogenic stimulus such as epidermal growth factor, basic fibroblast growth factor, and ROS, ERK cascade is activated and participates in the proliferation, survival, extracellular matrix synthesis, and immune regulation of trans‐differentiated HSCs.^43^ Wnt/β‐catenin pathway is complex and has a dual role in HSC activation based on specific biological context.^8^ During chronic liver fibrosis, upregulated Wnt/β‐catenin signaling promotes collagen deposition in activated HSCs.^2^ However, β‐catenin‐dependent canonical Wnt activation is needed to keep quiescent state of HSCs.^44^ TNFR1‐mediated NF‐κB signaling activation in HSCs inhibits the apoptosis and increases the survival of HSCs, which subsequently promotes fibrogenesis.^6^, ^45^ In this study, we identified a new target TGF‐β/Smad7 axis of CKI based on GSEA of RNA‐seq, which further widened and strengthened the preclinical knowledge of CKI.

The presence of myofibroblasts is a key common feature of chronic liver fibrosis, recognized as the major source of excess extracellular matrix molecules that have most abundant collagens.^8^ Nearly 82–96% of myofibroblasts were derived from HSCs in a fibrotic liver.^46^ Prolonged and repeated HSCs activation results in widespread scar formation, destructing normal liver function and architecture, and a failure of matrix degradation, which is mediated by inhibition of MMP degradative activity,^39^, ^47^ all these together make HSCs activation the central event of chronic liver fibrosis. Moreover, collagens produced from activated HSCs contribute epithelial–mesenchymal transition (EMT) to increase the risk of HCC formation.^3^ Hence, activated HSCs can be a crucial target for developing antifibrotic strategy.^8^ In this study, we found CKI intervention improved the liver function and architecture during chronic fibrosis. CKI decreased the expression of fibrogenic genes including αSMA and desmin to inhibit HSCs activation, and CKI reduced the extracellular matrix formation by downregulating collagen 1, fibronectin, and TIMP1 expression. These data provide evidence of CKI treatment in reversing activated HSCs to quiescent status, decreasing extracellular matrix deposition, and eventually inhibiting HCC.

TGF‐β is deemed to activate quiescent HSCs trans‐differentiate into a myofibroblast phenotype.^10^ Typically, TGF‐β binds to TGF‐β receptor and forms a complex to activate following Smad2/3 signaling.^48^ TGF‐β signaling involves all phases of the development of liver fibrosis and hepatocarcinogenesis.^10^ Activating TGF‐β signaling in HSCs promotes the extracellular matrix formation^6^ and promotes HCC growth.^49^ It is well accepted that important prior step of progression from chronic liver disease to HCC is the abrogation of cytostatic TGF‐β effects.^50^ Smad7 is recognized as a negative feedback regulator of TGF‐β signaling,^29^ and high expression of Smad7 in HCC as well as surrounding tissue has a link with increased overall survival.^50^ However, direct targeting of TGF‐β signaling by neutralization of TGF‐β isoforms or by inhibition of its receptors lead to unacceptable adverse effects.^11^, ^12^ In this study, CKI targeted Smad7 and upregulated its expression to induce TGFβR1 degradation and subsequently inhibited the phosphorylation of Smad2 and Smad3 to suppress TGF‐β/Smad2/3 signaling in activated HSCs, suggesting rebalanced TGF‐β/Smad7 signaling by CKI as a novel strategy in the treatment of liver fibrosis and oncogenesis.

**FIGURE 9:**
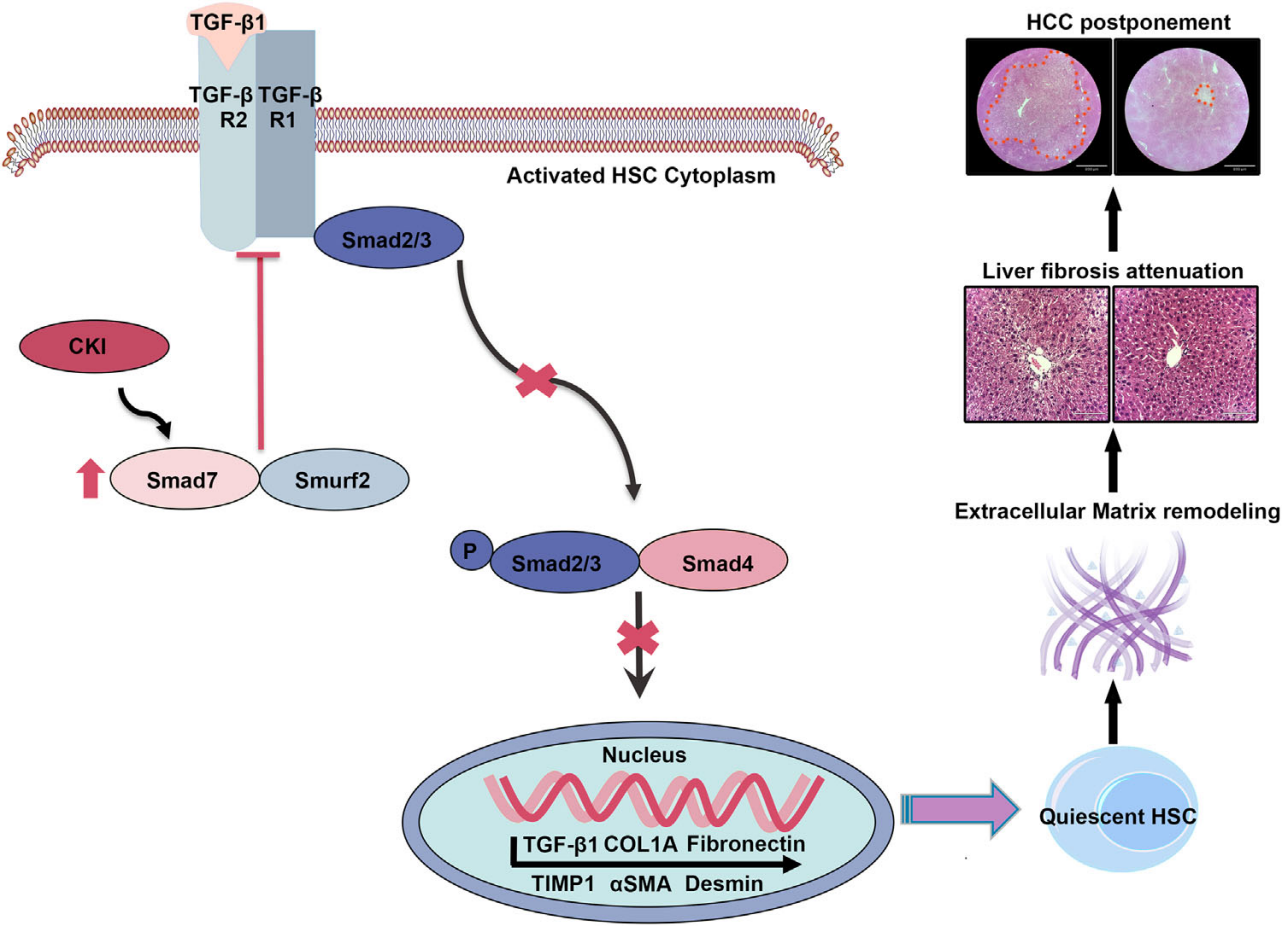
Schematic depiction of the antifibrosis function of CKI by rebalancing TGFβ/Smad7 signaling to inhibit HSCs activation. CKI targeted Smad7 and facilitated the interaction between Smad7 and TGF‐β receptor, which inhibited the phosphorylation of Smad2 and Smad3 to suppress the downstream TGFβ signaling. As a result, CKI prevented HSCs activation, subsequently interdicted extracellular matrix production to inhibit chronic liver fibrosis and finally postponed hepatocarcinogenesis

In conclusion, we conducted a meta‐analysis and preclinical study by using two mouse models to elucidate the novel antifibrosis function of CKI and investigated its underlying mechanism in liver fibrosis and HCC. Briefly, CKI targets Smad7 and facilitates the interaction between Smad7 and TGF‐β receptor to downregulate TGFβR1 expression and inhibits TGF‐β/Smad2/3 signaling in activated HSCs. As a result, CKI prevents HSCs activation, subsequently interdicts extracellular matrix production to inhibit chronic liver fibrosis progression and finally postpones hepatocarcinogenesis (Figure 8, 9). These data suggest the clinical utility of CKI as a promising candidate for preventing or treating liver fibrosis and preventing progression to HCC.

## CONFLICT OF INTEREST

The authors declare that there is no conflict of interest.

## ETHICS STATEMENT

All animals used in the present study were approved by the Animal Care and Use Committee of Shanghai Institute of Nutrition and Health, SIBS, CAS.

## AUTHOR CONTRIBUTIONS

Yang Yang, Qian Ba, Xiaoguang Li, and Hui Wang conceived and designed the experiments. Yang Yang, Qian Ba, Xiaoguang Li, and Hui Wang analyzed the data and wrote the manuscript. Hui Wang supervised the project. Yang Yang, Mayu Sun, Weida Li, Chaobao Liu, Zheshun Jiang, and Pengfei Gu performed the in vitro and in vivo studies. Jingquan Li, Wei Wang, and Rongli You provided writing assistance. All authors reviewed and approved the manuscript.

## Supporting information

Supporting Information

## Data Availability

All the data generated or analyzed during this study are included in this article.
