# Supplementary material for: Rebalancing TGF‐β/Smad7 signaling via Compound kushen injection in hepatic stellate cells protects against liver fibrosis and hepatocarcinogenesis
Source: Clin Transl Med. 2021 Jul 4;11(7):e410. doi: 10.1002/ctm2.410 (PMC8255064; doi:10.1002/ctm2.410)
Supplement: Supplementary file 1 — Supporting Information [file CTM2-11-e410-s001.pdf]

Supplementary information

Rebalancing TGF-β/Smad7 signaling via Compound kushen injection in hepatic stellate cells protects against liver fibrosis and hepatocarcinogenesis

Yang Yang[1,2#], Mayu Sun[1,2#], Weida Li[1#], Chaobao Liu[1], Zheshun Jiang[1], Pengfei Gu[1], Jingquan Li[1], Qian Ba[1*], Xiaoguang Li[1*], Hui Wang[1,2,*]

[1]*State Key Laboratory of Oncogenes and Related Genes, Center for Single-Cell Omics, School of Public Health, Shanghai Jiao Tong University School of Medicine, Shanghai, China.*

[2]*CAS Key Laboratory of Nutrition, Metabolism and Food safety, Shanghai Institute of Nutrition and Health, University of Chinese Academy of Sciences, Chinese Academy of Sciences, Shanghai, China*.

[*]**Correspondence:** huiwang@shsmu.edu.cn (H. Wang); lixg@shsmu.edu.cn (X. Li) and qba@shsmu.edu.cn (Q. Ba).

[#]Yang Yang, Mayu Sun and Weida Li contributed equally to this work.

Supplementary methods

The meta-analysis included in this study was performed in accordance with the Preferred Reporting Items for Systematic Reviews and Meta-Analyses (PRISMA) guidelines.

Database and Search strategy

The consulted electronic databases include PubMed, Web of Science, ClinicalTrials. gov, the China National Knowledge Infrastructure (CNKI), the Wanfang Database and the Cqvip Database. The last search date was April 10, 2021.

The search details were conducted with the terms “kushen injection” [Title/Abstract] OR “matrine injection” [Title/Abstract] OR “Sophora flavescens Injection” [Title/Abstract] AND “liver fibrosis” [Title/Abstract] OR “liver inflammation” [Title/Abstract] OR “HBV” [Title/Abstract] OR “HCV” [Title/Abstract] OR “NASH” [Title/Abstract] OR “liver cirrhosis” [Title/Abstract].

Inclusion and Exclusion criteria

Studies that met the following inclusion criteria were considered eligible for this meta-analysis: (1) trials were described as RCTs; (2) study patients were diagnosed as HBV, HCV or NASH induced chronic liver inflammation, fibrosis, or cirrhosis; (3) patients in the experimental group received CKI or CKI combined with regularly antiviral or liver fibrosis treatment agents, whereas patients in the control group only received antiviral or liver fibrosis treatment agents. (4) a minimum of two of the following outcomes were included in each study: ALT, AST, T-BIL, laminin (LN), hyaluronic acid (HA), procollagen type III (COL3) and collagen type IV (COL4).

The exclusion criteria were as follows: (1) the trials were not RCTs; (2) diagnostic criteria were not clearly reported in the trials; (3) relevant outcome indexes were not reported or estimated; (4) the detailed data regarding the liver functions were not available.

Data Extraction and Analysis

All identified articles were retrieved in full and the following data were extracted, including (1) publication information: the name of the first author and the publication year; (2) characteristics of patients: number, gender, age, the duration of disease and etiology; (3) Information on the intervention: dosage, duration and treatment cycle; (4) outcomes: the measured data on the efficiency of treatments. The characteristics of all included RCTs are summarized in Supplementary Table 5.

All analyses were performed using R software. Continuous data were shown as the standardized mean difference (SMD) with a 95% confidence intervals (CI) and the studies were pooled with the inverse variance weighted method under the fixed model and DerSimonian and Laird random-effects model. Heterogeneity was assessed using I^2 and τ^2 statistics. Substantial heterogeneity was considered when $I^2 > 50\%$ or $P < 0.01$. The included trials publication bias was evaluated by Rank correlation test of funnel plot asymmetry.

Supplementary Figures and Figure legends

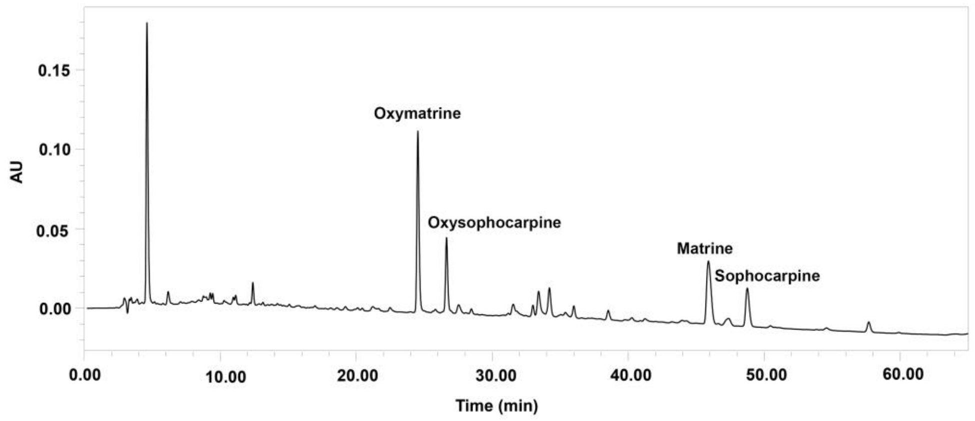

Figure S1 The HPLC fingerprint of CKI.

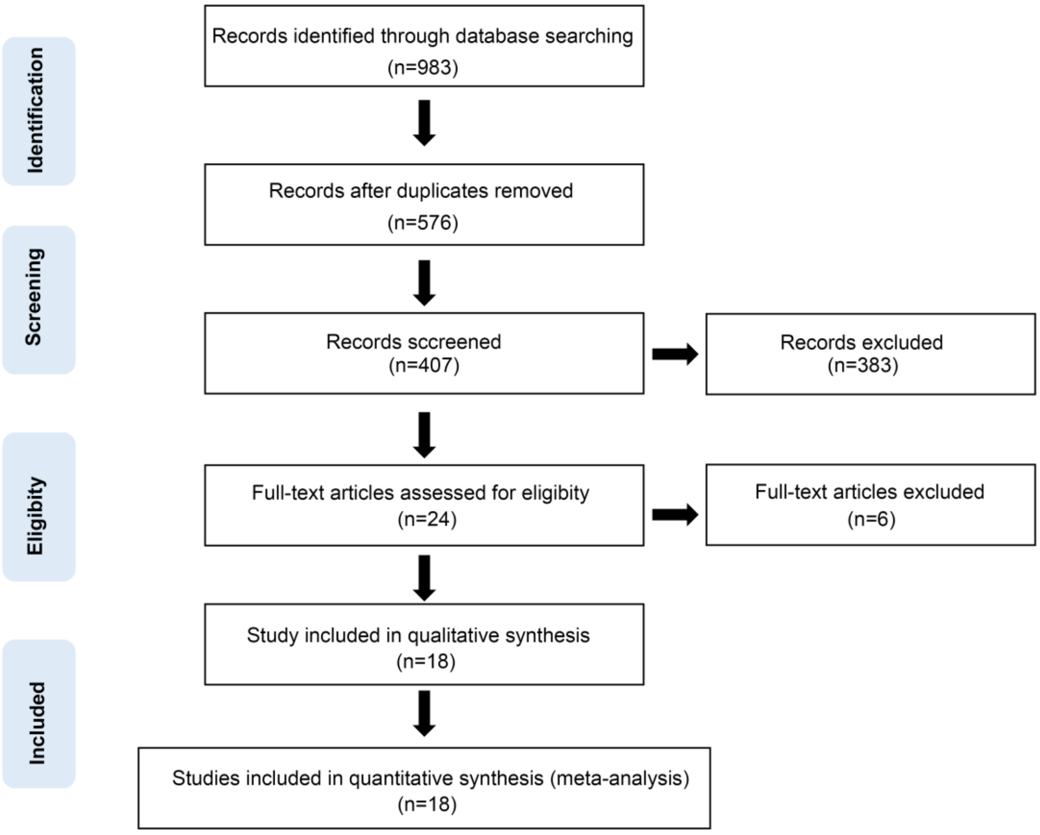

Figure S2 Working flow chart of the selection process for eligible studies.

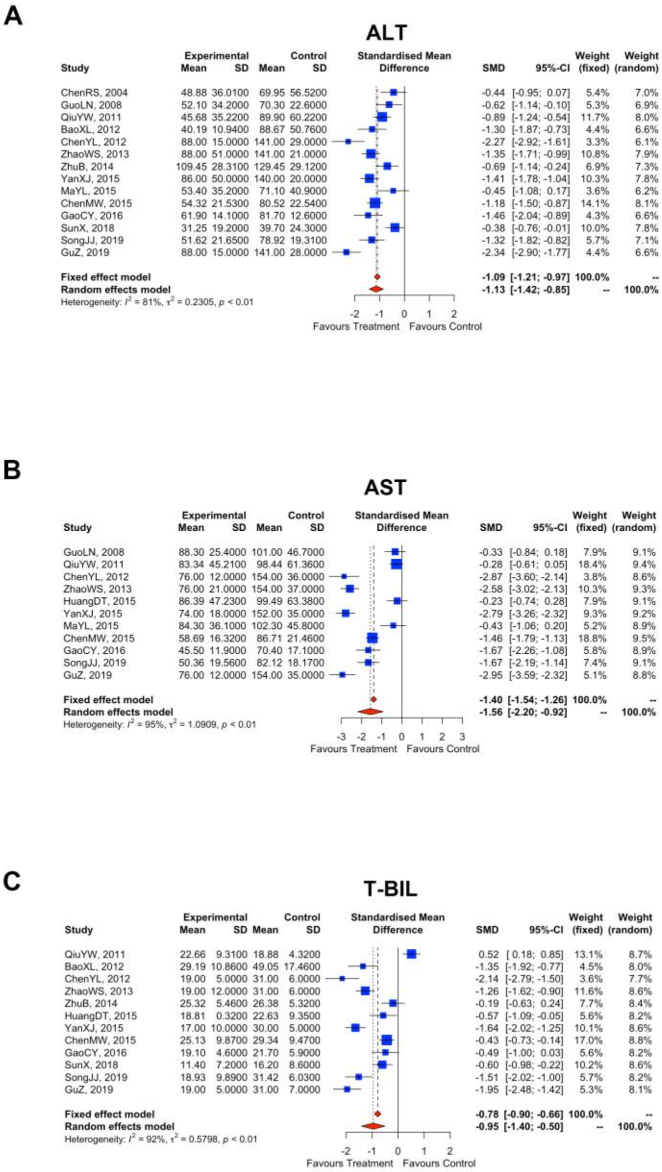

Figure S3 Forest plots showing a significant improvement in patients liver function after CKI intervention in the experimental group compared with control group, including the serum levels of (A) ALT, (B) AST and (C) T-BIL.

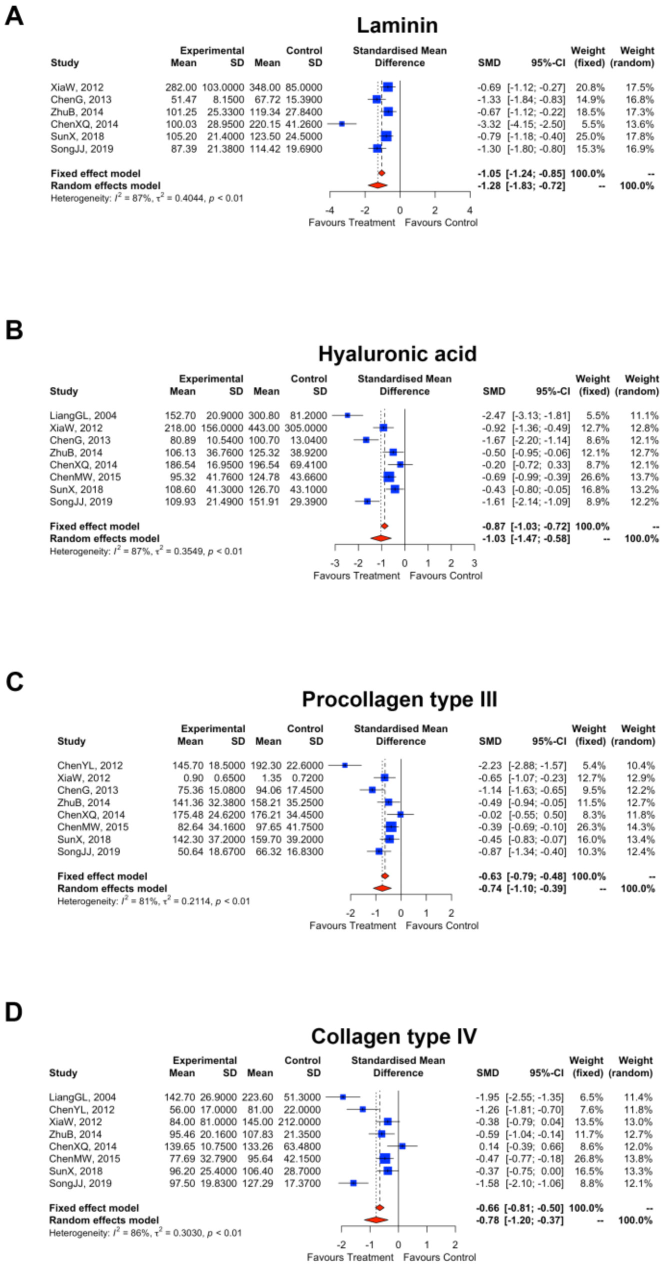

Figure S4 Forest plots showing a significant improvement in patients liver fibrosis after CKI intervention in the experimental group compared with control group, including the serum levels of (A) Laminin, (B) Hyaluronic acid, (C) Procollagen type III and (D) Collagen type IV.

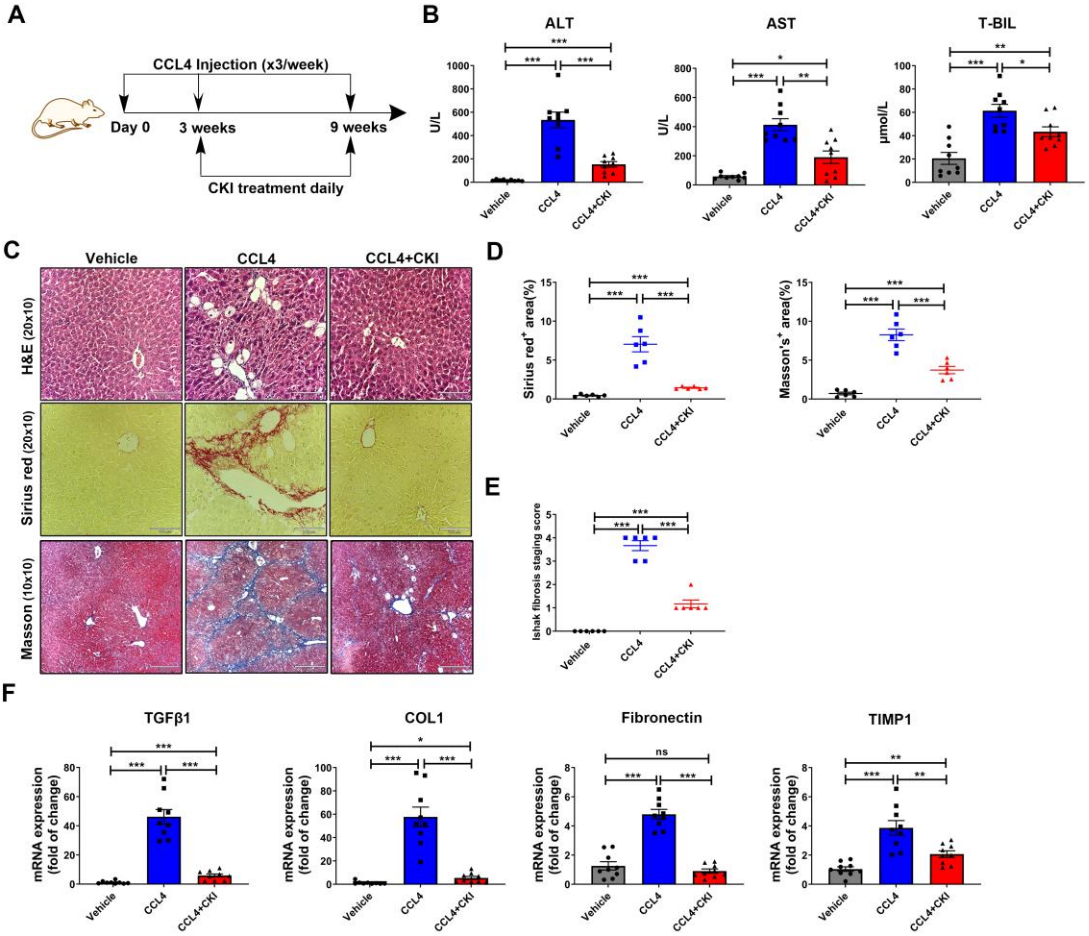

Figure S5. CKI attenuates chronic liver fibrosis.

(A) Scheme of experimental procedure for C57BL/6 mice intraperitoneally treated with 4mL/kg CCl_4 in olive oil for 9 weeks. Mice were intraperitoneally administrated with CKI (7.5 mL/kg) mouse for 6 weeks, starting at 3 weeks post initiation of CCl_4 challenge. (B) The serum levels of ALT, AST and T-BIL were detected after the final CKI treatment in CCl_4-treated mice (n=9). (C) Mice liver sections were collected for H&E (original magnification 20 x 10, scale bar, 110μm), Sirius red (original magnification 20 x 10, scale bar, 100μm), and Masson staining (original magnification 10 x 10, scale bar, 220μm) after final CKI treatment (n=6). (D) Positive Sirius red or Masson staining area were quantified by ImageJ analysis (n=6). (E) Ishak fibrosis score

of the Sirius red-stained liver sections (n=6). (F) The mRNA expression of *TGF-β1*, *COL1A*, *Fibronectin* and *TIMP1* were analyzed by qRT-PCR in mice liver tissues. Data are presented as means ± SEM. ns, *P*>0.05; *, *P* <0.05; **, *P*<0.01; ***, *P*<0.001.

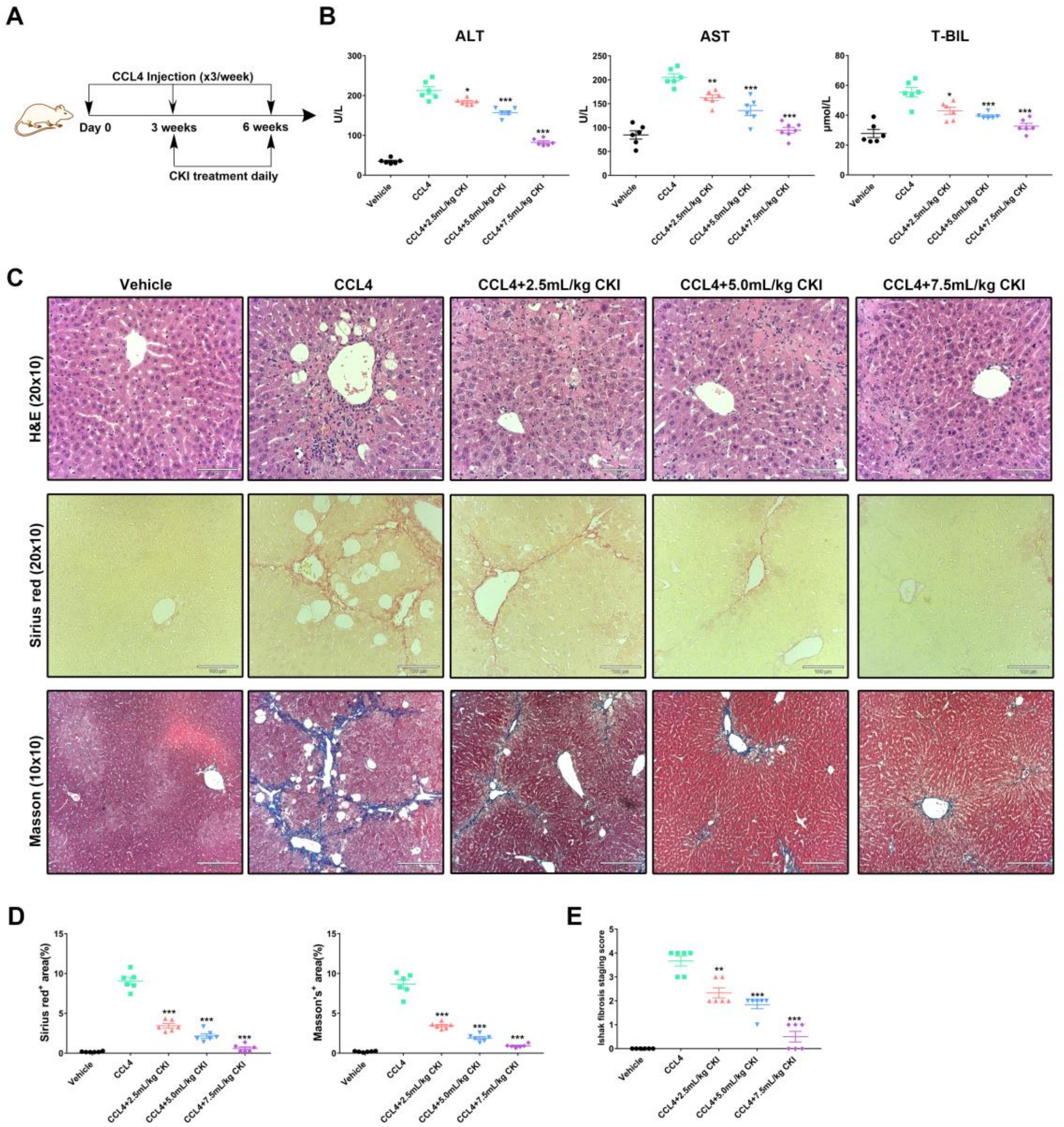

Figure S6. CKI inhibits chronic liver fibrosis in a dose-dependent manner.

(A) Scheme of experimental procedure for C57BL/6 mice intraperitoneally treated with 4mL/kg CCl_4 in olive oil for 6 weeks. Mice were intraperitoneally administrated with CKI (2.5, 5.0 and 7.5 mL/kg) for 3 weeks, starting at 3 weeks post initiation of CCl_4 challenge. (B) The serum levels of ALT, AST and T-BIL were detected after the final

CKI treatment in CCl_4-treated mice (n=6). (C) Mice liver sections were collected for H&E (original magnification 20 x 10, scale bar, 100μm), Sirius red (original magnification 20 x 10, scale bar, 100μm), and Masson staining (original magnification 10 x 10, scale bar, 210μm) after final CKI treatment (n=6). (D) Positive Sirius red or Masson staining area were quantified by ImageJ analysis (n=6). (E) Ishak fibrosis score of the Sirius red-stained liver sections (n=6). Data are presented as means ± SEM. ns, P>0.05; *, P <0.05; **, P<0.01; ***, P<0.001.

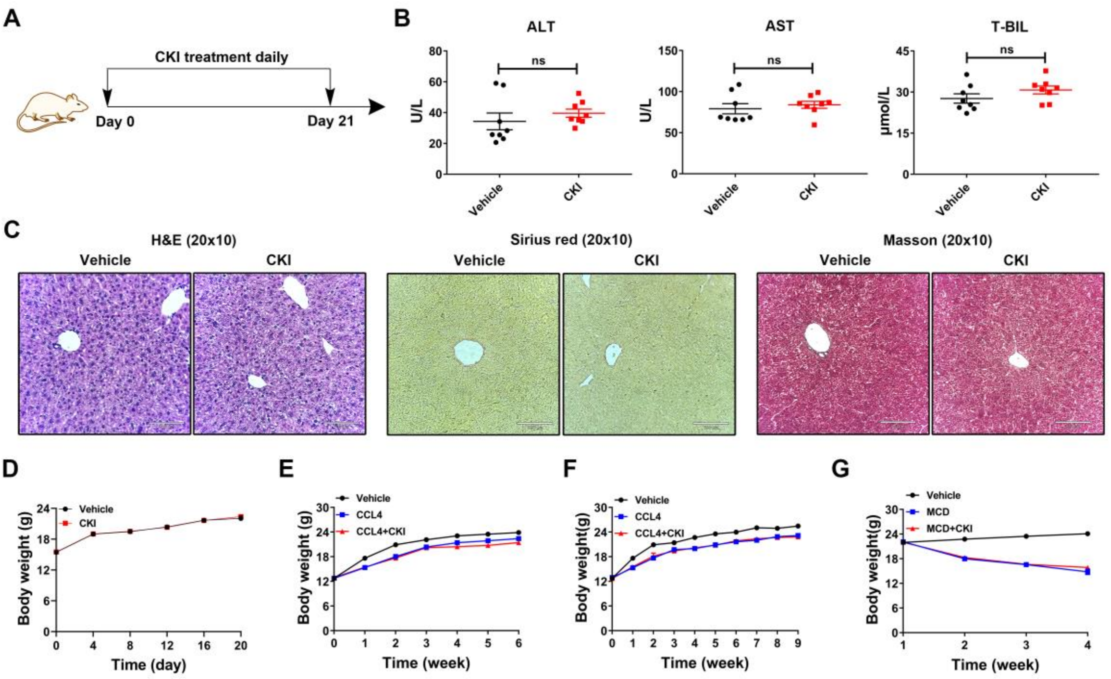

Figure S7. CKI treatment has no side effect on mice.

(A) Scheme of experimental procedure for normal C57BL/6 mice intraperitoneally administrated with CKI (7.5 mL/kg) for 3 weeks. (B) The serum levels of ALT, AST and T-BIL were detected after the final CKI treatment (n=8). (C) Mice liver sections were collected for H&E, Sirius red, and Masson staining (original magnification 20 x 10, scale bar, 100μm). (D-G) The curves of mouse bodyweight changes were shown

from different models. (D) related to the model in Supplementary Figure 6A. (E) related to the model in Figure 1A. (F) related to the model in Supplementary Figure 4A. (G) related to the model in Figure 1C. Data are presented as means ± SEM. ns, *P*>0.05.

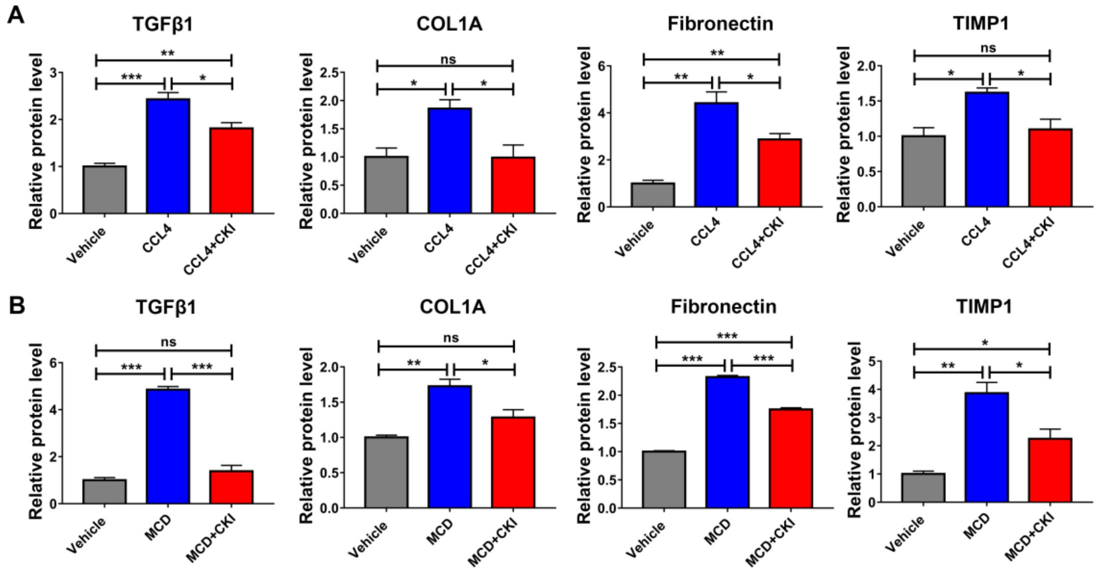

Figure S8. Protein quantification Related to Figure 1K and L.

(A and B) Quantitative analysis of the protein expression of TGF-β1, COL1A, Fibronectin and TIMP1 in liver tissues from CCl_4-challenged (A) or MCD diet-challenged (B) mice. Data are presented as means ± SEM. ns, *P*>0.05; *, *P* <0.05; **, *P*<0.01; ***, *P*<0.001.

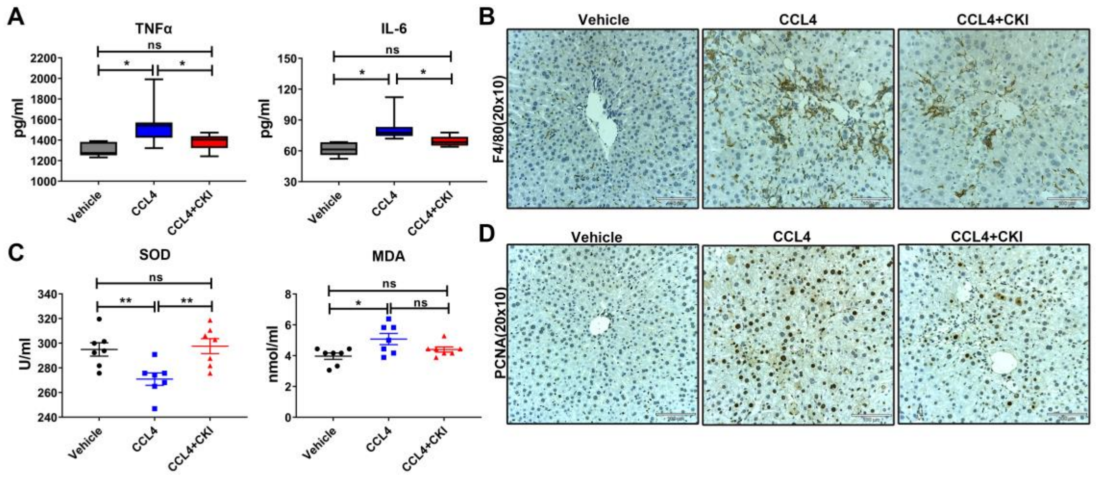

Figure S9. CKI ameliorates inflammatory response, oxidative stress, and liver compensatory proliferation of mice in Supplementary Figure 5A model.

(A) The serum levels of TNFα and IL-6 were detected by Elisa assays (n=9). (B) Representative F4/80 immunostaining of macrophages in mice liver sections were displayed (original magnification 20 x 10, scale bar, 100μm). (C) The serum levels of SOD and MDA were quantified by Elisa assays (n=7). (D) Representative immunostaining of PCNA in the liver section of mice were shown after indicated treatments (original magnification 20 x 10, scale bar, 100μm). Data are presented as means ± SEM. ns, *P*>0.05; *, *P* <0.05; **, *P*<0.01; ***, *P*<0.001.

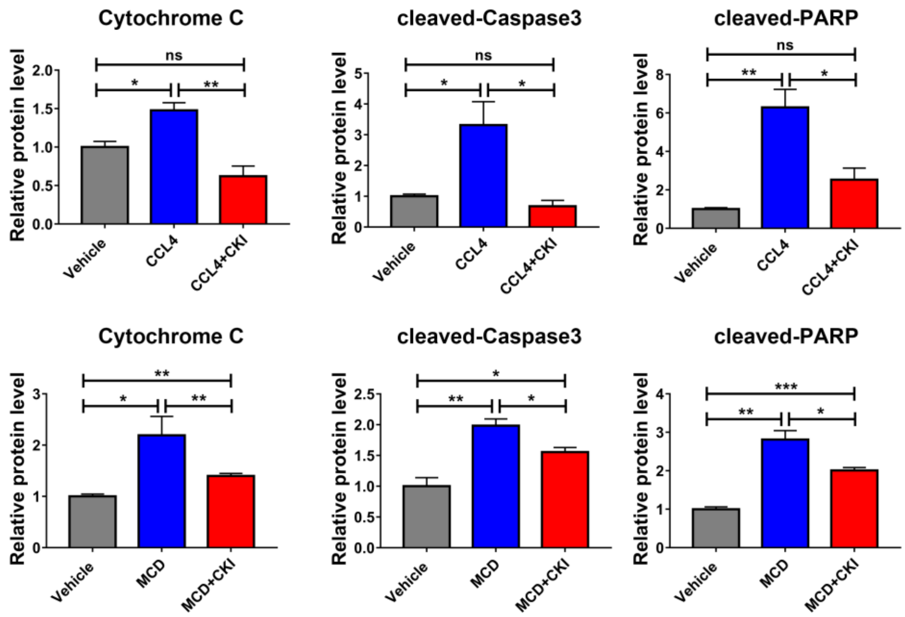

Figure S10. Protein quantification Related to Figure 2G.

Quantitative analysis of the protein expression of Cytochrome C, cleaved-Capase3 and cleaved-PARP in liver tissues from CCl_4-challenged or MCD diet-challenged mice. Data are presented as means ± SEM. ns, *P*>0.05; *, *P* <0.05; **, *P*<0.01; ***, *P*<0.001.

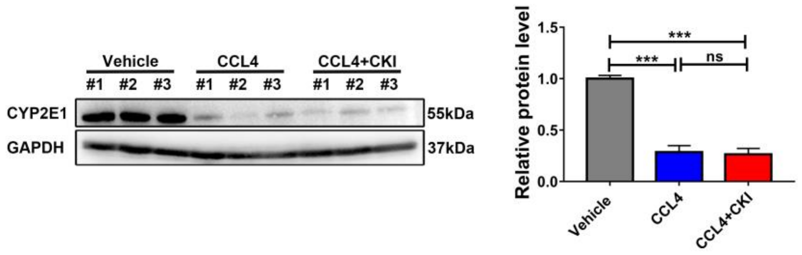

Figure S11. CKI treatment has no effect on CCl_4 metabolism *in vivo*.

Mice were intraperitoneally administrated with CKI (7.5 mL/kg) mouse for 6 weeks, starting at 3 weeks post initiation of CCl_4 challenge. Western bolt analysis of the expression of CYP2E1 in mouse liver lysates.

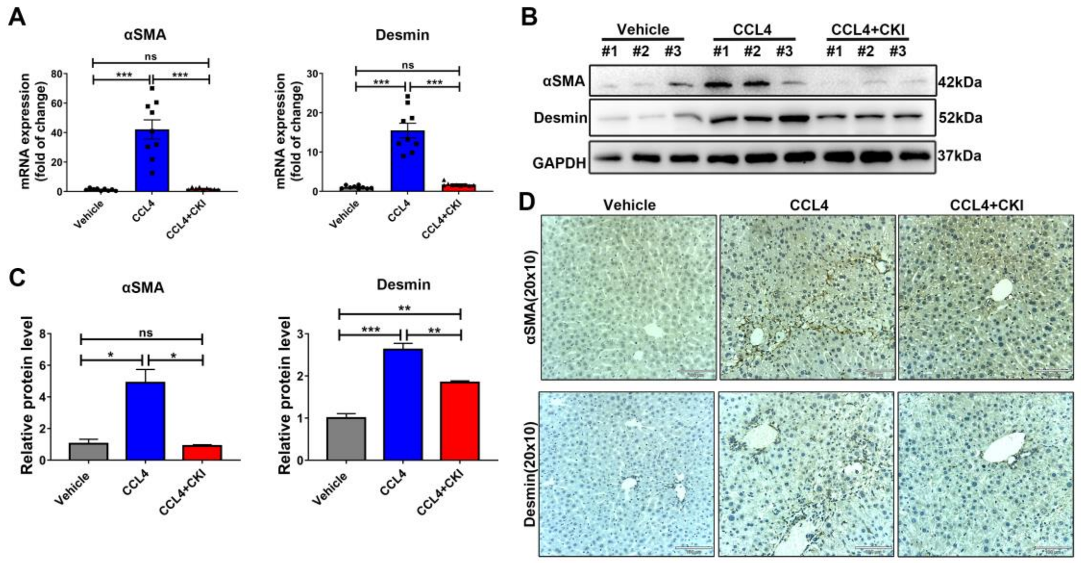

Figure S12. CKI inhibits liver fibrosis by suppressing HSCs activation in vivo from Supplementary Figure 5A model.

(A) Mice were treated with CCl_4 for 9 weeks along with CKI treatment for 6 weeks (n=9). The mRNA expression of *αSMA* and *desmin* in mice liver tissues were analyzed by qRT-PCR. (B) Western blot assay for detecting the expression of αSMA and desmin in mice liver tissues. (C) Quantitative analysis of the protein expression of αSMA and desmin. (D) Representative immunohistochemistry images of αSMA and desmin stain

of liver sections after indicated treatments (original magnification 20 x 10, scale bar, 100μm). Data are presented as means ± SEM. ns, *P*>0.05; *, *P* <0.05; **, *P*<0.01; ***, *P*<0.001.

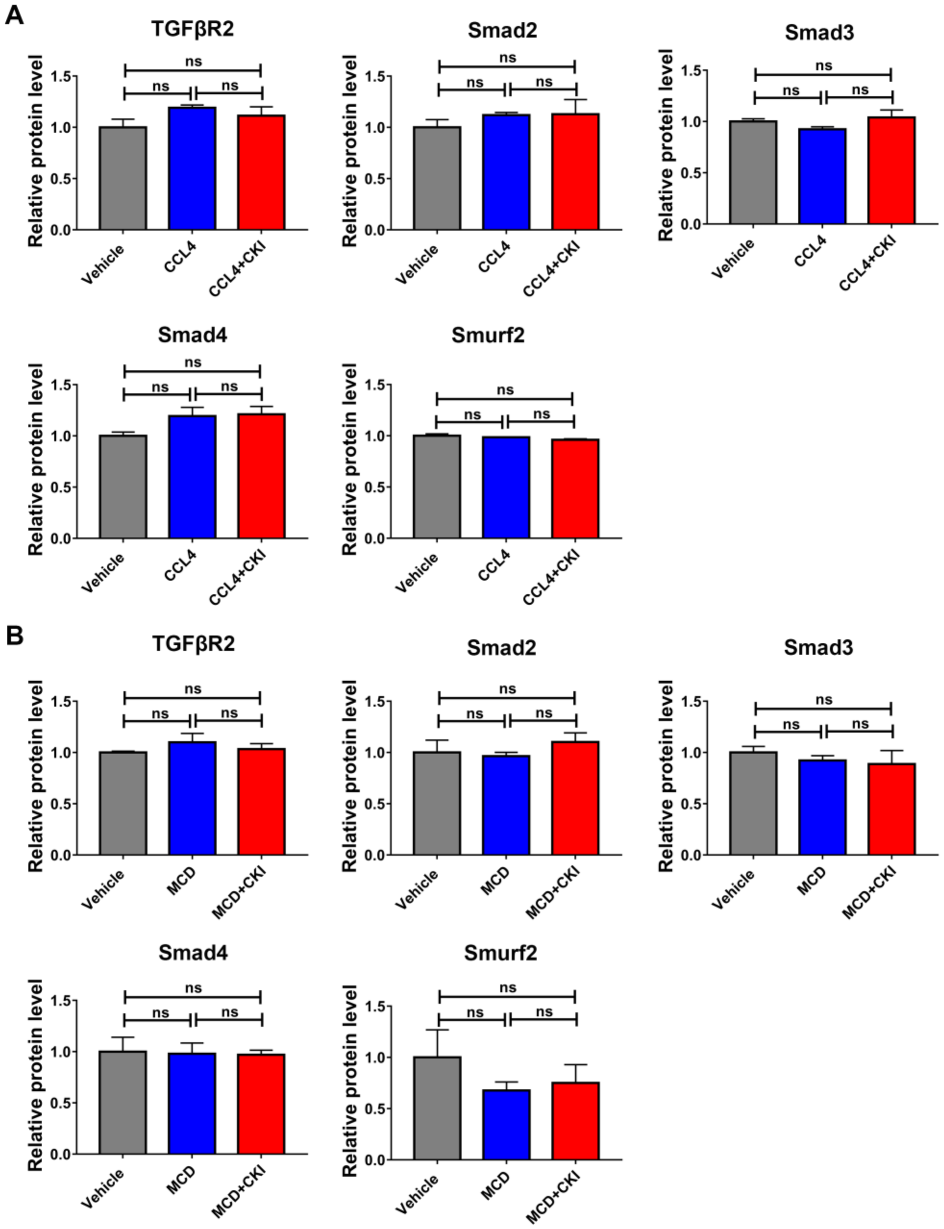

Figure S13. Protein quantification Related to Figure 4B and D.

(A and B) Quantitative analysis of the protein expression of TGFβ2, Smad2, Smad3, Smad4 and Smurf2 in liver tissues from CCl_4-challenged (A) or MCD diet-challenged (B) mice. Data are presented as means ± SEM. ns, *P*>0.05.

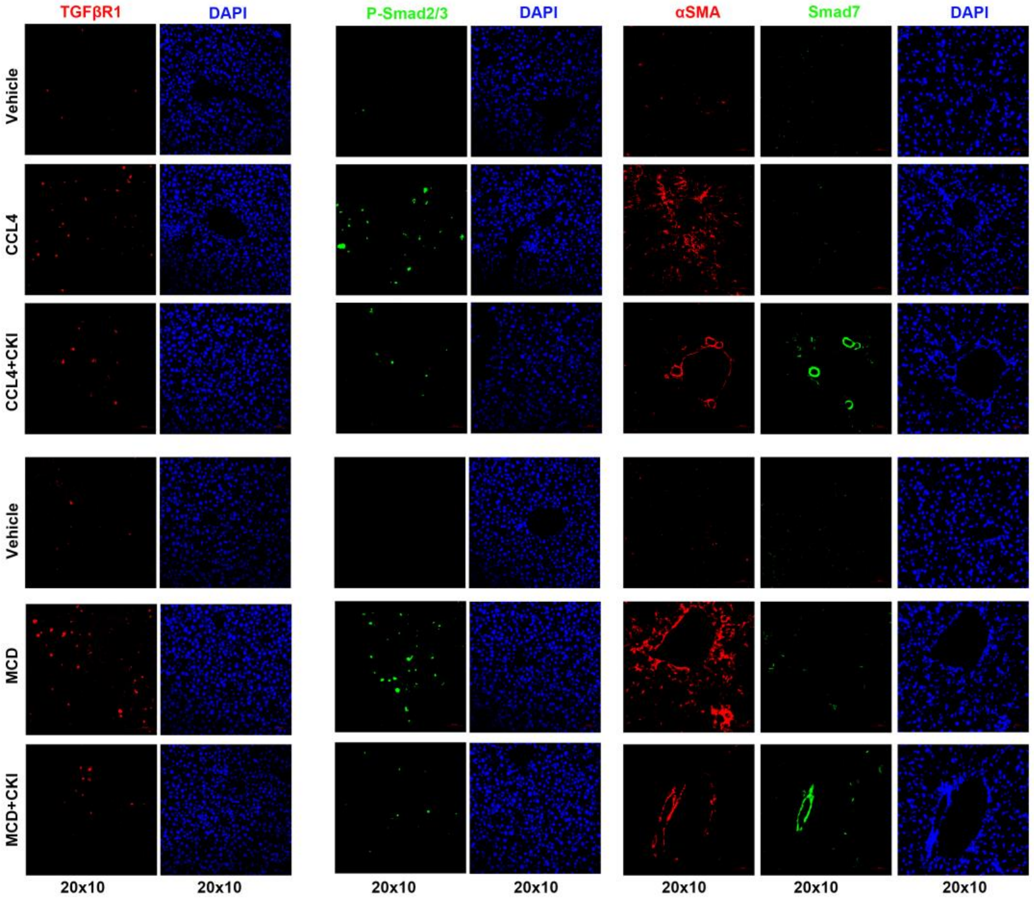

Figure S14. The uncropped immunofluorescence staining images of TGFβR1, p-Smad2/3, αSMA, Smad7 and DAPI for Figure4.

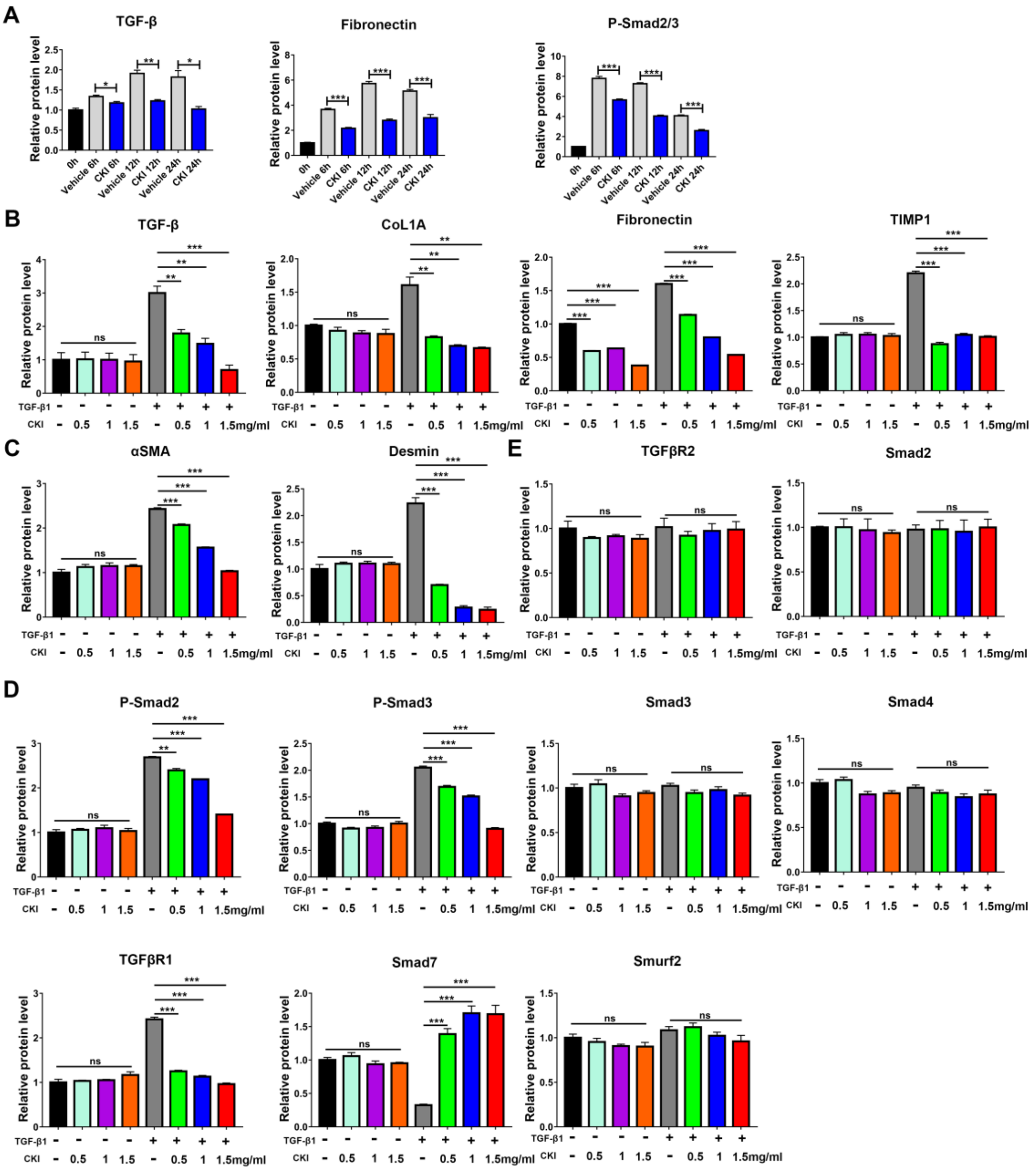

Figure S15. Protein quantification Related to Figure 5C, D, G and H.

(A) Quantitative analysis of the protein expression of TGFβ, Fibronectin and p-Smad2/3 in LX-2 cells from Figure 5C. (B) Quantitative analysis of the protein expression of TGFβ, COL1A, Fibronectin and TIMP1 protein levels in LX-2 cells from Figure 5B. (C) Quantitative analysis of the protein expression of αSMA and desmin in LX-2 cells from Figure 5G. (D and E) Quantitative analysis of the protein expression of TGFβR1, TGFβR2, p-Smad2, total Smad2, p-Smad3, total Smad3, Smad4, Smad7

and Smurf2 in LX-2 cells from Figure 5H. Data are presented as means ± SEM. ns, *P*>0.05; *, *P* <0.05; **, *P*<0.01; ***, *P*<0.001.

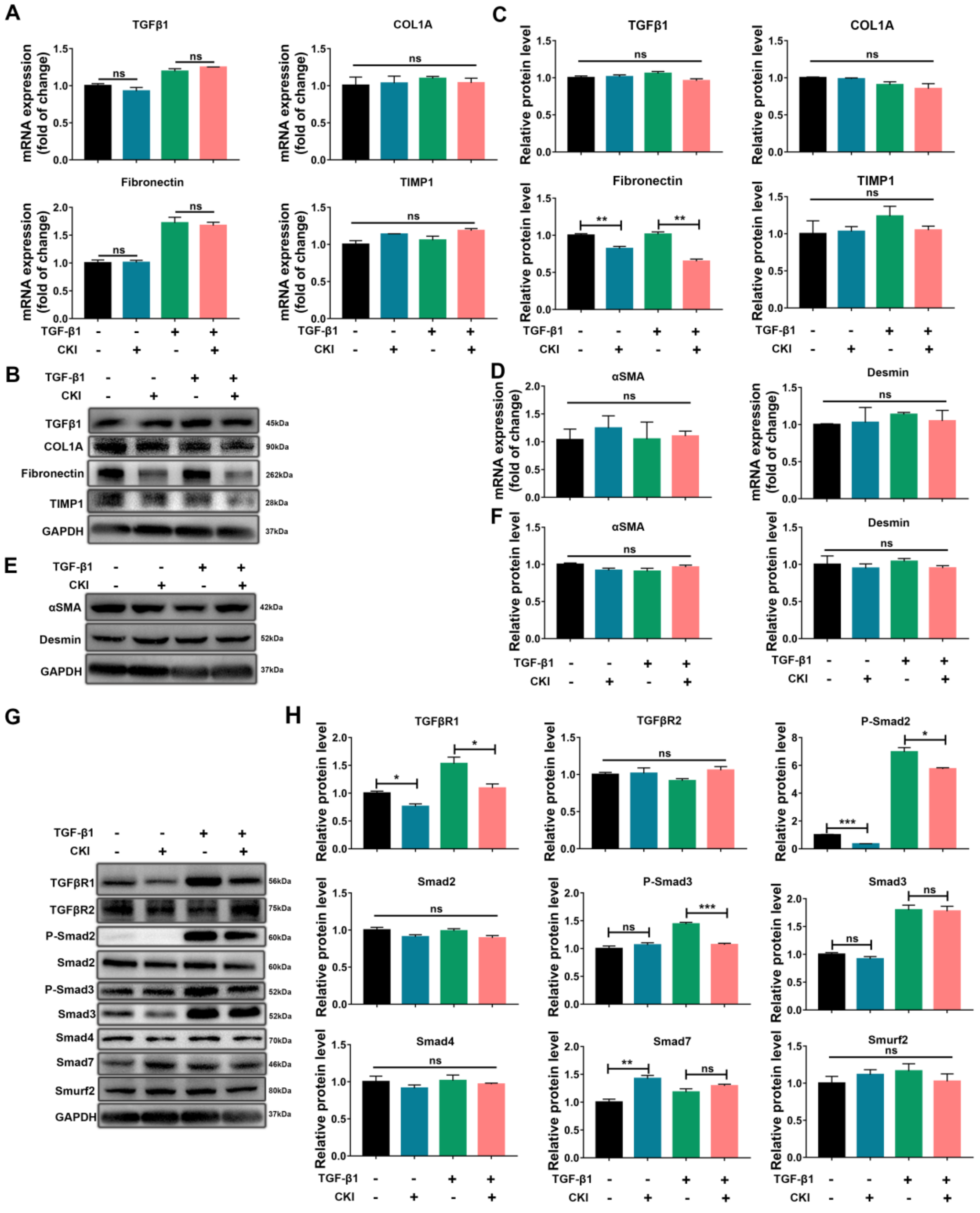

Figure S16. The influence of CKI on LO2 cells *in vitro*.

(A) LO2 cells were treated with 5ng/mL TGF-β1 along with 1mg/mL CKI for12h. The mRNA expression of *TGF-β1*, *COL1A*, *Fibronectin* and *TIMP1* were detected by qRT-

PCR in LO2 cell lysates. (B) Western bolt for TGF-β1, COL1A, Fibronectin and TIMP1 in LO2 cells after CKI treatment. (C) Quantitative analysis of the protein expression of TGF-β1, COL1A, Fibronectin and TIMP1. (D) qRT-PCR analysis of *αSMA* and desmin *mRNA* expression in LO2 cells. (E) The protein expression of αSMA and desmin were quantified by western blot in LO2 cells. (F) Quantitative analysis of the protein expression of αSMA and desmin. (G) Western blot analysis of TGFβR1, TGFβR2, p-Smad2, total Smad2, p-Smad3, total Smad3, Smad4, Smad7 and Smurf2 in LO2 cells. (H) Quantitative analysis of the protein expression of TGFβR1, TGFβR2, p-Smad2, total Smad2, p-Smad3, total Smad3, Smad4, Smad7 and Smurf2. Data are presented as means ± SEM. ns, $P>0.05$; *, $P<0.05$; **, $P<0.01$; ***, $P<0.001$.

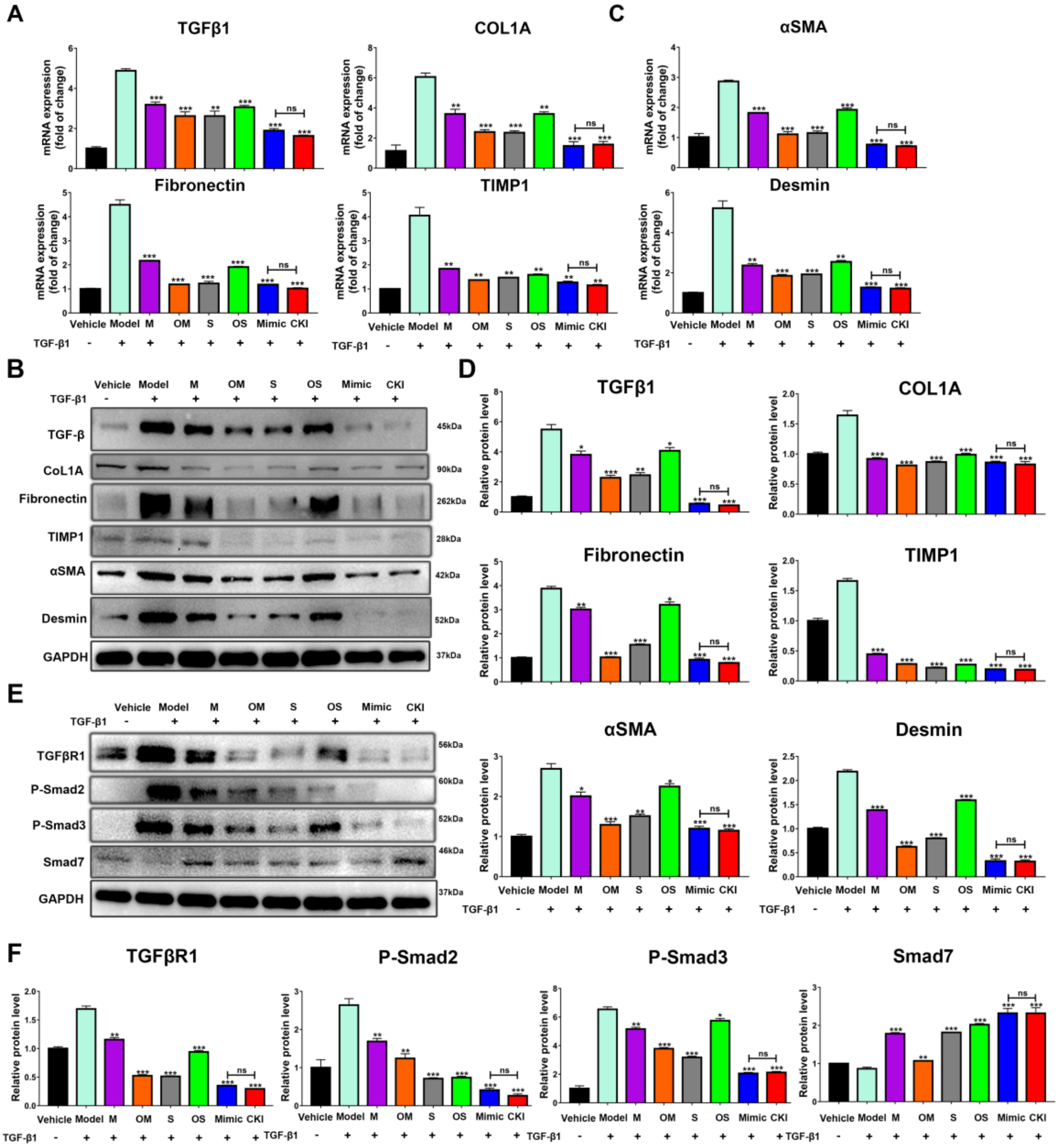

Figure S17. The material basis responsible for the antifibrosis effect of CKI.

(A) LX-2 cells were treated with or without 5ng/mL TGF-β1 along with Matrine (M, 0.3034mg/mL), Oxymatrine (OM, 0.964mg/mL), Sophocarpine (S, 0.0827mg/mL), OxySophocarpine (OS, 0.229mg/mL), Mimic (M+OM+S+OS) and CKI (1.5mg/mL) for 12h. The mRNA expression of *TGF-β1*, *COL1A*, *Fibronectin*, and *TIMP1* were detected by qRT-PCR in LX-2 cell lysates. (B) The protein expression of TGFβ, COL1A, Fibronectin, TIMP1, αSMA and desmin were quantified by western blot in

LX-2 cells. (C) qRT-PCR analysis of HSCs activation markers *αSMA* and *desmin* mRNA expression in LX-2 cells. (D) Quantitative analysis of the protein expression of TGFβ, COL1A, Fibronectin, TIMP1, αSMA and desmin. (E) Western blot analysis of TGFβR1, p-Smad2, p-Smad3 and Smad7 in LX-2 cells. (F) Quantitative analysis of the protein expression of TGFβR1, p-Smad2, p-Smad3 and Smad7. Data are presented as means ± SEM. ns, *P*>0.05; **, *P*<0.01; ***, *P*<0.001.

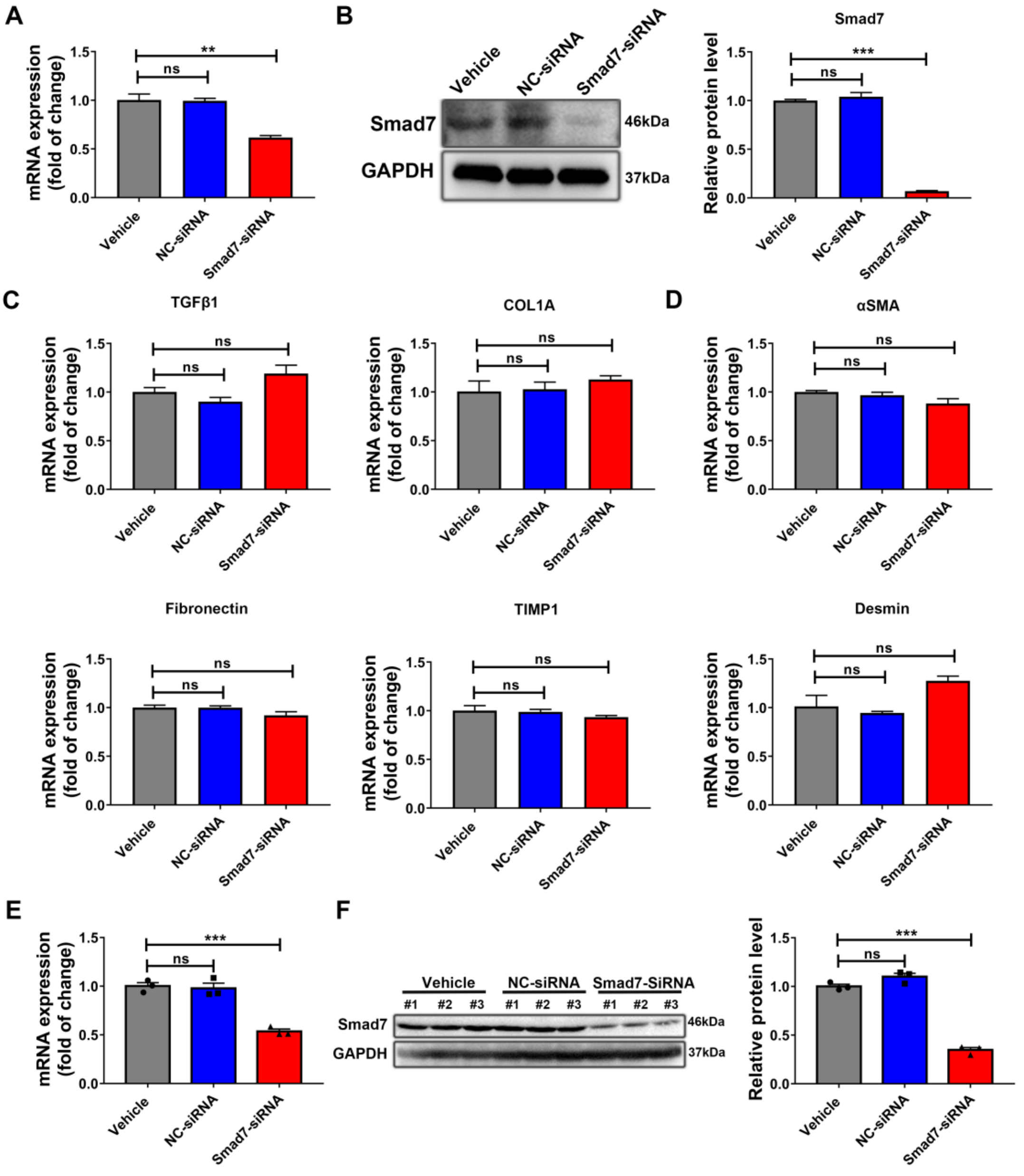

Figure S18. The knock-down efficiency of Smad7-siRNA *in vitro* and *in vivo*.

(A and B) The mRNA and protein levels of Smad7 in LX-2 cells after Smad7-siRNA treatment. (C) Smad7 siRNA-knockdown has no effect on the mRNA expression of *TGF-β1*, *COL1A*, *Fibronectin* and *TIMP1* in quiescent LX-2 cells. (D) Smad7 siRNA-knockdown didn't influence the activation of quiescent LX-2 cells *in vitro*. (E and F) 72h after 5mg/kg Smad7-siRNA treatment, the knockdown efficiency of Smad7-siRNA in mouse livers were explored by qRT-PCR and western blotting (n=3). Data are presented as means ± SEM. ns, P>0.05, **, P<0.01; ***, P<0.001.

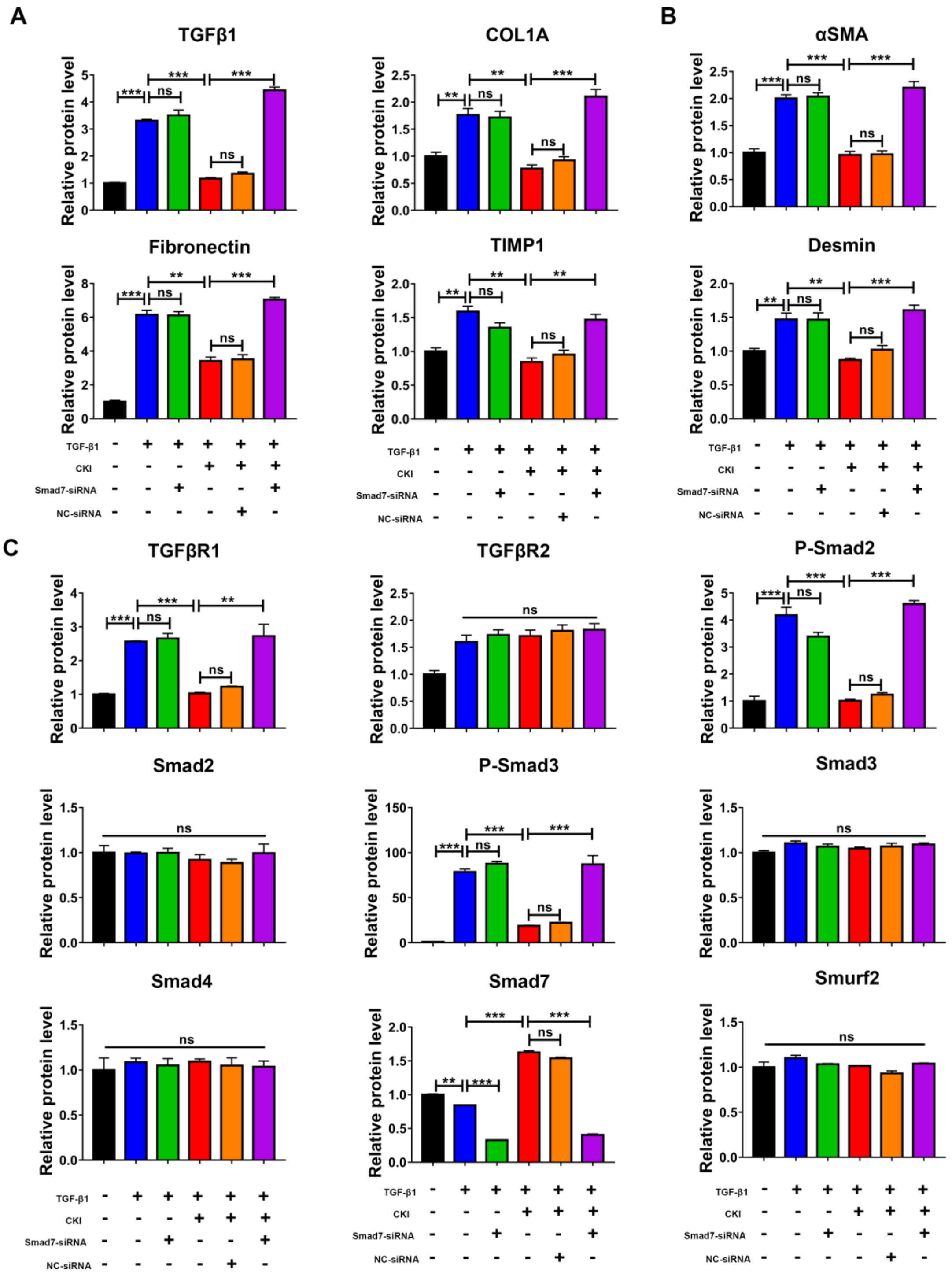

Figure S19. Protein quantification Related to Figure 6C, E and F.

(A) Quantitative analysis of the protein expression of TGFβ, COL1A, Fibronectin and TIMP1 from Figure 6C. (B) Quantitative analysis of the protein expression of αSMA and desmin from Figure 6E. (C) Quantitative analysis of the protein expression of

TGFβR1, TGFβR2, p-Smad2, total Smad2, p-Smad3, total Smad3, Smad4, Smad7 and Smurf2 from Figure 6F. Data are presented as means ± SEM. ns, *P*>0.05; **, *P*<0.01; ***, *P*<0.001.

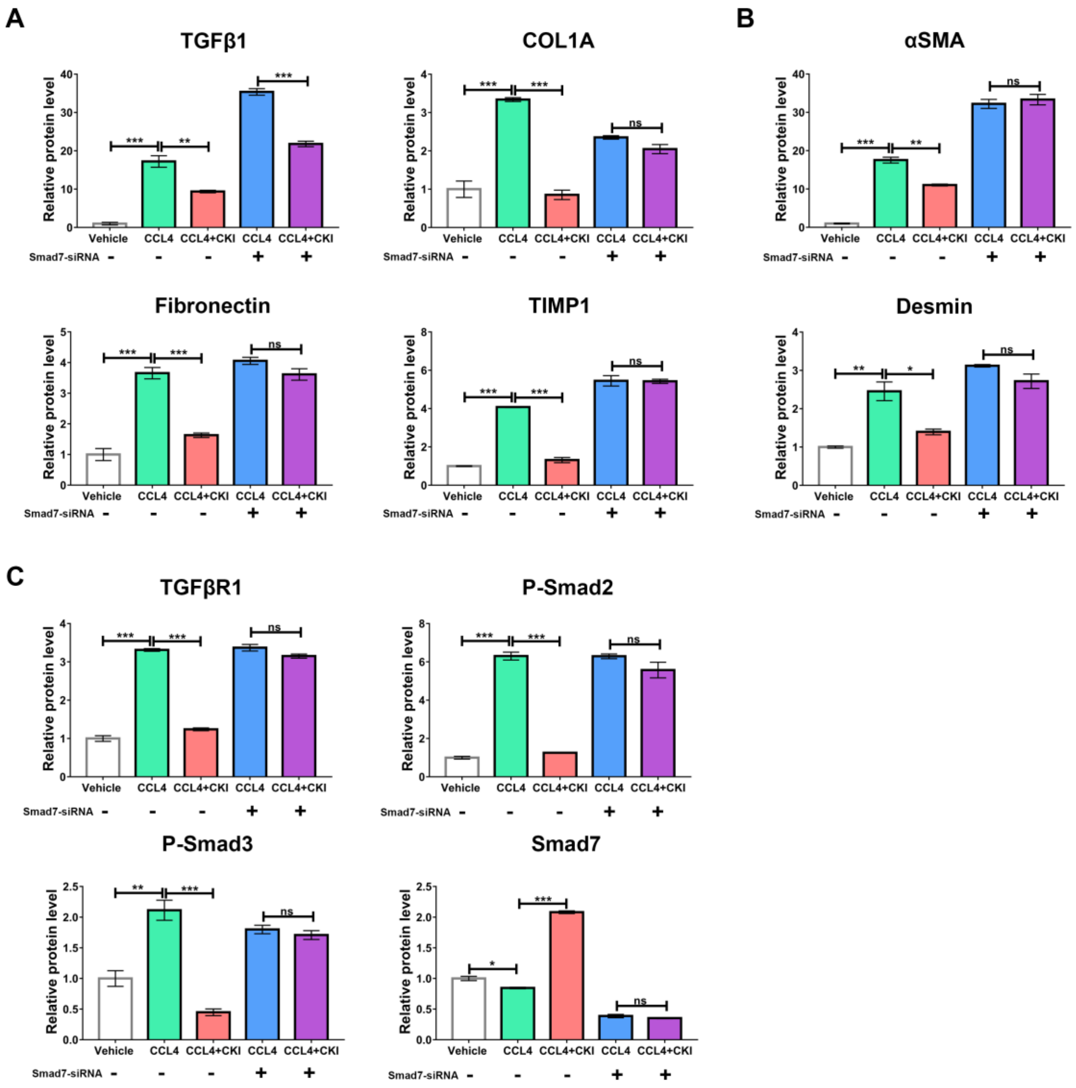

Figure S20. Protein quantification Related to Figure 7G, I and J.

(A) Quantitative analysis of the protein expression of TGF-β1, COL1A, Fibronectin and TIMP1 from Figure 7G. (B) Quantitative analysis of the protein expression of αSMA and desmin in Figure 7I. (C) Quantitative analysis of the protein expression of

TGFβR1, p-Smad2, p-Smad3 and Smad7 from Figure 7J. Data are presented as means ± SEM. ns, *P*>0.05; *, *P* <0.05; **, *P*<0.01; ***, *P*<0.001.

Supplementary tables

Table S1. The identified primary bioactive alkaloid content in CKI by HPLC

Alkaloid	Molecular formula	Concentration
Oxymatrine	$C_{15}H_{25}O_2N_2$	9.640mg/ml
Oxysophocarpine	$C_{15}H_{23}O_2N_2$	2.290mg/ml
Matrine	$C_{15}H_{25}ON_2$	3.034mg/ml
Sophocarpine	$C_{15}H_{23}ON_2$	0.827mg/ml

Table S2. Mouse primer sequences for real-time PCR

Targets		Primer sequence (5'-3')
GAPDH	Forward	ATGTTCCAGTATGACTCCACTCAC
	Reverse	GACACAGTAGACTCCACGACATA
β-actin	Forward	AGAGGGAAATCGTGCGTGAC
	Reverse	CAATAGTGATGACCTGGCCGT
TGF-β1	Forward	GGTTCATGTCATGGATGGTGC
	Reverse	TGACGTCACTGGAGTTGTAC
CoL1A	Forward	GGAAACCTCTCTCGCCTCTT
	Reverse	GAACAGGGTGTTCCTGAGA
Fibronectin	Forward	GGCTACATCATCCGCCATCA
	Reverse	GCCCGGATTAAGGTTGGTGA
TIMP1	Forward	GGCTAAATTCATGGGTTCAC
	Reverse	CTCAGAGTACGCCAGGGAACCA
	Reverse	AGTTGCTTCTAGCCCAAAGAAC
αSMA	Forward	CATCACCAACTGGGACGACA
	Reverse	TCCGTTAGCAAGGTCGGATG
Desmin	Forward	GTGGATGCAGCCACTCTAGC
	Reverse	TTAGCCGCGATGGTCTCATAC
Smad7	Forward	GGCCGGATCTCAGGCATTC
	Reverse	TTGGGTATCTGGAGTAAGGAGG

Table S3. Human primer sequences for real-time PCR

Targets	Primer sequence (5’-3’)	
GAPDH	Forward	AAGAAGG TGGTGAAGCAGGC
	Reverse	TCCACCACCCT GTTGCTGTA
Actin	Forward	GTTGTCGACGACGAGCG
	Reverse	GCACAGAGCCTCGCCTT
TGF-β1	Forward	CAATTCCTGGCGATACCTCAG
	Reverse	GCACAACTCCGGTGACATCAA
CoL1A	Forward	TGGCCAAGAAGACATCCCTGAAGT
	Reverse	ACATCAGGTTTCCACGTCTCACCA
Fibronectin	Forward	CCATCGCAA ACCGCTGCCAT
	Reverse	AACACTTCTCAGCTATGGGCTT
TIMP1	Forward	CTTCTGCAATTCCGACCTCGT
	Reverse	ACGCTGGTATAAGGTGGTCTG
αSMA	Forward	ACTG AGCGTGGCTATTCCTCCGTT
	Reverse	GCAGTGGCCATCTCATTTTCA
Desmin	Forward	GACGTGGATGCAGCTACTCTA
	Reverse	GGAACGCGATCTCCTCGTTG
Smad7	Forward	GGACAGCTCAATTCGGACAAC
	Reverse	GTACACCCACACACCATCCAC

Table S4 Primary antibodies information

Product code	Name	Dilution range	Company
ab13575	Cytochrome C	1:1000	Abcam
9661	cleaved-Caspase3	1:1000	CST
9544	cleaved-PARP	1:1000	CST
3711	TGF-β	1:1000	CST
sc-59772	COL1A	1:200	Santa Cruz
ab2413	Fibronectin	1:1000	Abcam
sc-21734	TIMP1	1:200	Santa Cruz
ab5694	αSMA	1:1000	Abcam
ab15200	Desmin	1:1000	Abcam
ab28146	CYP2E1	1:1000	Abcam
ab31013	TGF-β R1	1:1000	Abcam
sc-17702	TGF-β R2	1:200	Santa Cruz
sc-365846	Smad7	1:200	Santa Cruz
8828	P-Smad2/3	1:1000	CST
3108	P-Smad2	1:1000	CST
5339	Smad2	1:1000	CST
9520	P-Smad3	1:1000	CST
9523	Smad3	1:1000	CST
38454	Smad4	1:1000	CST
12024	Smurf2	1:1000	CST
2118	GAPDH	1:1000	CST
7074	Anti-rabbit IgG HRP-linked	1:2000	CST
7076	Anti-mouse IgG HRP-linked	1:2000	CST
5127	Anti-rabbit IgG (conformation specific)	1:2000	CST
sc-516102	m-IgGκ BP-HRP	1:2000	Santa Cruz

Table S5 Characteristics of the included trials

Study ID	Sample size (M/F)		Age		Progress (year)		Etiology
	Control	Treatment	Control	Treatment	Control	Treatment	
Song J. 2019	22/16	21/17	43.34±3.9	43.15±3.65	3.66±0.71	3.79±0.54	HBV induced liver inflammation
Chen R. 2004	17/13	19/11	16-59	17-56	NR		HBV induced liver inflammation
Sun X. 2018	30/25	29/26	44.15±11.82	43.95±12.37	2.40±1.57	2.34±1.62	HBV induced liver inflammation
Zhu B. 2014	48/32		42.15±9.31		10.32±3.12		HBV induced liver inflammation
Zhao W. 2013	52/20	53/19	40±6	41±5	6±2.5	6±2.5	HBV induced liver inflammation
Qiu Y. 2011	43/27	42/28	16-56	18-59	NR		HBV induced liver inflammation
Yan X. 2015	76/64		42.15±8.98		NR		HBV induced liver inflammation
Bao X. 2014	38/20		40 ± 9.1		NR		HBV induced liver cirrhosis
Guo L. 2008	21/9	18/12	20-56	18-60	NR		HCV induced liver inflammation
Ma Y. 2015	12/8	11/9	36.5±0.5	38.2±0.5	NR		HCV induced liver inflammation
Gao C. 2016	20/10	19/11	52.6±8.9	54.1±7.7	2.6±1	2.8±0.8	HCV induced liver inflammation
Chen Y. 2012	35/25		52.2±7.6	53.9±6.9	NR		HBV or HCV induced liver cirrhosis
Chen M, 2015	56/34	51/39	56.78±12.32	52.31±12.56	NR		HBV or HCV induced liver cirrhosis
Gu Z, 2019	23/18	25/16	55.5±6.4	54.2±3.2	4.44±1.06	4.53±1.09	HBV induced liver cirrhosis
Chen G, 2013	45/30		41.05±9.64		NR		HBV induced liver cirrhosis
Xia W, 2012	35/10	32/13	21-53	25-53	NR		HBV induced liver fibrosis
Liang G, 2004	23/9	22/10	36.1±12.8	38±13.3	6.8±4.8	7.6±4.3	HBV induced liver fibrosis
Chen X, 2014	16/12	16/12	40.1±5.9	40.4±6.2	4.8±1.6	4.9±1.1	HCV induced liver cirrhosis

Table S6 Intervention characteristics and outcome measures of the included trials

Study ID	Intervention (Day) Control	Intervention (Day) Treatment	Duration	Outcomes measures
Song J. 2019	Entacvir	CKI, 20mL, iv + Entacvir	8 weeks	ALT, AST, T-BIL, LN, HA, COL3, COL4
Chen R. 2004	Diammonium glycyrrhizinate	CKI, 20mL, iv + Diammonium glycyrrhizinate	8 weeks	ALT, AST, T-BIL
Sun X. 2018	Interferon α-1b	CKI, 10mL, iv + Interferon α-1b	6 weeks	ALT, T-BIL, LN, HA, COL3, COL4
Zhu B. 2014	Adefovir dipivoxil	CKI, 10mL, iv + Adefovir dipivoxil	8 weeks	ALT, T-BIL, LN, HA, COL3, COL4
Zhao W. 2013	Entacvir	CKI, 20mL, iv + Entacvir	8 weeks	ALT, AST, T-BIL
Qiu Y. 2011	Lamivudine	CKI, 20mL, iv + Lamivudine	8 weeks	ALT, AST, T-BIL
Yan X. 2015	Lamivudine	CKI, 20mL, iv + Lamivudine	6 weeks	ALT, AST, T-BIL
Bao X. 2014	Hepatinica	CKI, 600mg, iv + Hepatinica	8 weeks	ALT, T-BIL
Guo L. 2008	Interferon injection	CKI, 20mL, iv + Interferon injection	12 weeks	ALT, AST
Ma Y. 2015	Interferon injection	CKI, 20mL, iv + Interferon injection	3 months	ALT, AST
Gao C. 2016	Hepatinica	CKI, 20mL, iv + Bicyclol	6 months	ALT, AST, T-BIL
Chen Y. 2012	Hepatinica	CKI, 20mL, iv + Hepatinica	8 weeks	ALT, AST, T-BIL, COL3, COL4
Chen M, 2015	Hepatinica	CKI, 600mg, iv + Hepatinica	6 months	ALT, AST, T-BIL, HA, COL3, COL4
Gu Z, 2019	Alprostadil Injection	CKI, 600mg, iv + Alprostadil Injection	21 days	ALT, AST, T-BIL
Chen G, 2013	Lamivudine	CKI, 20mL, iv + Lamivudine	12 months	LN, HA, COL3
Xia W, 2012	Compound glycyrrhizin	CKI, 30mL, iv + Compound glycyrrhizin	8 weeks	LN, HA, COL3, COL4
Liang G, 2004	Thymosin injection	CKI, 20mL, iv	8 weeks	HA, COL4
Chen X, 2014	Hepatinica	CKI, 600mg, iv + Hepatinica	6 months	LN, HA, COL3, COL4

Table S7 List of selected studies for meta-analysis

1. Song J, Song S. Observation on the therapeutic effect of Entecavir combined with Compound kushen injection against chronic Hepatitis B. Journal of Qiqihar Medical University. 2019; 40(5):598-599. DOI: 10.3969/j.issn.1002-1256.2019.05.030
2. Chen R. Compound kushen injection combined with Arixin injection in the treatment of 30 patients with chronic Hepatitis B. Journal of Practical Traditional Chinese Medicine. 2004; 20(9):500.
3. Sun X, Wang H, Gong Y, Liu Q, Xiao L. Compound kusGhen injection and Interferon α-1b in Treating 55 Cases of Chronic Hepatitis B. Western Journal of Traditional Chinese Medicine. 2018; 31(12):95-97.
4. Zhu B, Zhang P. Observation on therapeutic effect of Compound kushen injection combined with adefovir dipivoxil in the treatment for the chronic hepatitis B. The Chinese Journal of Clinical Pharmacology. 2014; 30(3):179-181. DOI: 10.13699/j.cnki.1001-6821.2014.03.005
5. Zhao W, Li Y, Ji J. The improvement effect of patient liver function with the chronic hepatitis B after Compound kushen injection combined with Entecavir. Chinese Journal of Information on Traditional Chinese Medicine. 2013; 20(3):70-71. DOI: 10.3969/j.issn.1005-5304.2013.03.027
6. Qiu Y, Sun H, Yu C. Compound kushen injection combined with Lamivudine in treating 70 cases of chronic hepatitis B. Zhejiang Journal of Traditional Chinese Medicine. 2011; 46(5):389.
7. Yan X, Dong L, Zhang L. Observation on Therapeutic Effect of Compound Kushen Injection Combined with Lamivadine in Treatment of Chronic Hepatitis B. Research of Integrated Traditional Chinese and Western Medicine. 2015; 7(1):7-9. DOI: 10.3969/j.issn.1674-4616.2015.01.002
8. Bao X, Yin G. On Effect of Matrine Injection in Treatment of Hepatitis B Cirrhosis and Its Mechanism. Journal of Beihua University (Natural Science). 2014; 15(6):780-782. DOI: 10.11713/j.issn.1009-4822.2014.06.020
9. Guo L, Sun J, Wang G. Compound kushen injection combined with Interferon in treating 30 cases of chronic hepatitis C. Modern Journal of Integrated Traditional

Chinese and Western Medicine. 2008; 17(36):5582.

10. Ma Y, Zhang L. The therapeutic effect of Compound kushen injection combined with Interferon against chronic hepatitis C. Asia-Pacific Traditional Medicine. 2015; 11(13):114-115. DOI: 10.11954/ytctyy.201513057.
11. Gao C, Yang Y. Compound kushen injection combined with Bicyclol in treating 30 cases of chronic hepatitis C. Shaanxi Medical Journal. 2016; 45(9):1239-1241. DOI: 10.3969/j.issn.1000-7377.2016.09.066
12. Chen Y, Lin J, Lou X, Yan H, Chen J. The Curing Effect of Complex Radix Sophorae Flavescentis Injection on Hepatitis Liver Cirrhosis. 2012; 12(5):53-55. DOI: 10.3969/j.issn.1671-332X.2012.5.021
13. Chen M. Influence of Kushen injection on fibrosis index and interleukin in serum and ascites for patients with cirrhosis. Journal of Hainan Medical University. 2015; 21(4): 476-478. DOI: 10.13210/j.cnki.jhmu.20150104.006.
14. Gu Z. The therapeutic effect of Compound kushen injection combined with Alteplase against hepatitis liver cirrhosis. 2019; 31(6):108-109.
15. Chen G, Mao J, Jiao Z, Li Y. Efficacy Observation of Compound Sophora flavescens Injection Combined with Lamivadine on Chronic Hepatitis B Cirrhosis. China Pharmacy. 2013; 24(4): 342-344. DOI: 10.6039/j.issn.1001-0408.2013.04.19
16. Xia W, Wang D, Xiao S, Shan J. Efficacy of Compound kushen Injection in Preventing Hepatic Fibrosis Formation in Patients with Chronic Hepatitis B. Research of Integrated Traditional Chinese and Western Medicine. 2012; 4(5): 229-230+233. DOI: 10.3969/j.issn.1674-4616.2012.05.002
17. Liang G. Compound kushen injection in treating 30 cases of chronic hepatitis C induced liver fibrosis. Guangxi Medical Journal. 2004; 26(11):1628-1630.
18. Chen X. Clinical efficacy of Compound Matrine injection in treatment of hepatitis C cirrhosis and its mechanism. Drugs & Clinic. 2014; 29(5):527-531. DOI: 10.7501/j.issn.1674-5515.2014.05.019

Table S8 The *P* value of risk of publication bias

Index	*P* value
ALT	0.622
AST	0.392
T-BIL	0.100
LN	0.039
HA	0.138
COL3	0.083
COL4	0.083
